# Interferon-Responsive Genes Are Targeted during the Establishment of Human Cytomegalovirus Latency

**DOI:** 10.1128/mBio.02574-19

**Published:** 2019-12-03

**Authors:** Elizabeth G. Elder, Benjamin A. Krishna, James Williamson, Eleanor Y. Lim, Emma Poole, George X. Sedikides, Mark Wills, Christine M. O’Connor, Paul J. Lehner, John Sinclair

**Affiliations:** a Department of Medicine, University of Cambridge, Cambridge, United Kingdom; b Lerner Research Institute, Cleveland Clinic, Cleveland, Ohio, USA; c Cambridge Institute for Medical Research, University of Cambridge, Cambridge, United Kingdom; Brown University

**Keywords:** IFI16, US28, viral latency, cytomegalovirus, interferon response

## Abstract

Human cytomegalovirus (HCMV) latency is an active process which remodels the latently infected cell to optimize latent carriage and reactivation. This is achieved, in part, through the expression of viral genes, including the G-protein-coupled receptor US28. Here, we use an unbiased proteomic screen to assess changes in host proteins induced by US28, revealing that interferon-inducible genes are downregulated by US28. We validate that major histocompatibility complex (MHC) class II and two pyrin and HIN domain (PYHIN) proteins, myeloid cell nuclear differentiation antigen (MNDA) and IFI16, are downregulated during experimental latency in primary human CD14^+^ monocytes. We find that IFI16 is targeted rapidly during the establishment of latency in a US28-dependent manner but only in undifferentiated myeloid cells, a natural site of latent carriage. Finally, by overexpressing IFI16, we show that IFI16 can activate the viral major immediate early promoter and immediate early gene expression during latency via NF-κB, a function which explains why downregulation of IFI16 during latency is advantageous for the virus.

## Abstract (precis)

Human cytomegalovirus (HCMV) is a ubiquitous herpesvirus which infects 50 to 100% of humans worldwide. HCMV causes a lifelong subclinical infection in immunocompetent individuals but is a serious cause of mortality and morbidity in the immunocompromised and neonates. In particular, reactivation of HCMV in the transplant setting is a major cause of transplant failure and related disease. Therefore, a molecular understanding of HCMV latency and reactivation could provide insights into potential ways to target the latent viral reservoir in at-risk patient populations.

## INTRODUCTION

Lifelong persistence of human cytomegalovirus (HCMV) is underpinned by viral latency and reactivation. Following primary infection, the ubiquitous betaherpesvirus HCMV establishes latency in cell types, including early myeloid lineage cells (1–4). The viral genome is maintained in these cells in the relative absence of immediate early (IE) gene expression or production of infectious virions. Reactivation of HCMV is associated with differentiation of myeloid lineage cells to mature dendritic cells and macrophages; as such, reactivation events are thought to occur sporadically throughout the lifetime of the host (5–8). In immunocompetent individuals, both primary infection and reactivation events are well controlled by a broad and robust immune response (9). However, HCMV reactivation is a major cause of morbidity and mortality in immunocompromised patients, including stem cell and organ transplant recipients (10, 11).

A key hallmark of latency is the relative suppression of IE gene expression (1, 2, 12–14), which is controlled by the major immediate early promoter (MIEP) (15–17), and the subsequent lack of infectious virion production. The establishment of latency via MIEP repression in early myeloid lineage cells requires both host and viral factors (18). One viral factor that suppresses MIEP activity is the G-protein-coupled receptor (GPCR) US28, a virally encoded chemokine receptor homologue, which is expressed *de novo* during latency as well as being delivered to cells with the incoming virion (19–24), and this incoming viral US28 is functional (25). US28 modulates the signaling pathways of early myeloid cells; it attenuates mitogen-activated protein (MAP) kinases, NF-κB, and c-*fos*, while activating STAT3 and inducible nitric oxide synthase (iNOS) (21, 23, 25). All of these contribute to the repression of MIEP activity. This US28-mediated signaling is so critical to latency that US28-deleted viruses, or the loss of G-protein coupling by the US28 mutant R129A, results in lytic infection of undifferentiated myeloid cells (19, 21, 23, 25). Furthermore, when examined, these US28-mediated effects on cell signaling did not occur during lytic infection or in permissive cells (21), implying that US28 represses the MIEP during latency but does not impair reactivation following cellular differentiation. This is reflective of the cell type-specific nature of US28-mediated signaling (21, 26).

Since US28 can modulate all these pathways and control the MIEP, we hypothesized that US28 would also cause changes in host protein expression. Here, we perform a proteomic screen comparing host cell protein abundance in myelomonocytic THP-1 cells expressing wild-type US28 (US28-WT) or the US28-R129A signaling mutant. We find that the expression of many host proteins are decreased in the presence of US28-WT compared with the US28-R129A mutant and that a large proportion of these proteins are interferon inducible. In particular, the two pyrin and HIN domain (PYHIN) family proteins, myeloid cell nuclear differentiation antigen (MNDA) and gamma interferon (IFN-γ)-inducible protein 16 (IFI16), are downregulated by US28, as well as major histocompatibility complex (MHC) class II proteins. IFI16 is associated with the nuclear sensing of herpesvirus DNA (27–31) and control of herpesvirus gene expression (32–39) and also represses viral transcription during HIV latency (40), but the effects of IFI16 on HCMV latent infection are unknown. Downregulation of HLA-DR/MHC class II is important for the evasion of CD4^+^ T cell responses to latently infected myeloid cells (41), while antiviral effects of MNDA have yet to be reported.

We have validated the downregulation of IFI16, MNDA, and HLA-DR in US28-expressing THP-1 cells and during experimental latency in primary CD14^+^ monocytes. We find that HCMV downregulates IFI16 within the first 24 h of infection of myeloid cells in a US28-dependent manner, but this effect is lost in differentiated dendritic cells. We propose that downregulation of IFI16 is beneficial to the establishment of latency because overexpression of IFI16 drives MIEP activity and IE gene expression via NF-κB. By targeting the downregulation of IFI16, US28 actively promotes the establishment of latency in early myeloid lineage cells.

## RESULTS

### Proteomic analysis reveals US28-induced changes in host proteins in myeloid cells.

**FIG 1:**
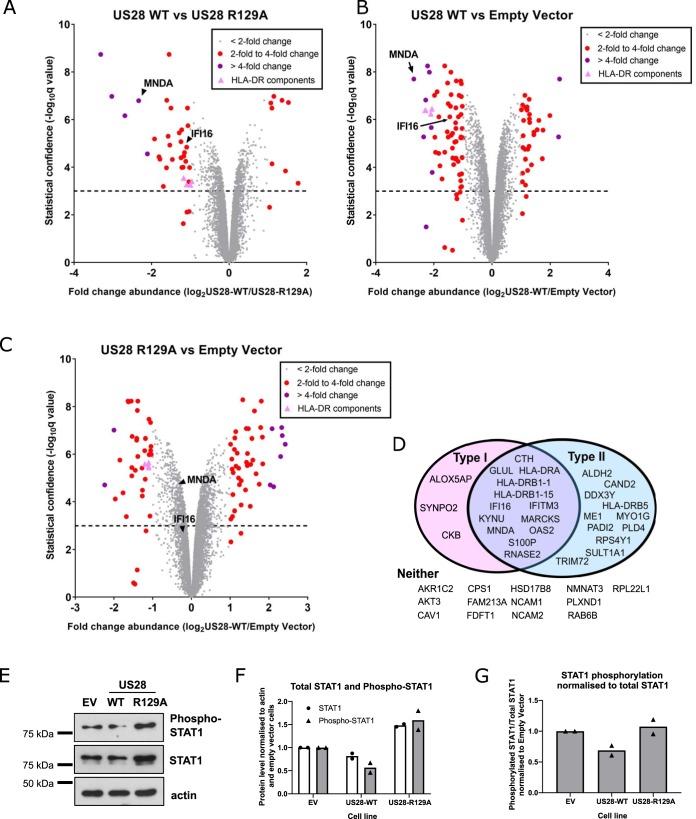
US28 induces changes in the host proteome of THP-1 cells. (A to C) THP-1 cells expressing empty vector, US28-WT, and US28-R129A were subjected to total cell proteomic analysis using a TMT labeling approach as described in Materials and Methods. Each circle represents one human protein and is shown in gray if its abundance changes by a factor of less than 2, in red if between 2- and 4-fold, and in purple if greater than 4-fold. The exception is components of the HLA-DR complex, which are represented by pink triangles. The dotted line represents a significance threshold of a *q* value of 0.001; a *q* value of < 0.001 is considered significant. *q* values of significance between groups were calculated by Benjamini-Hochberg correction of *P* values generated using the moderated T-test LIMMA within the R environment. Comparison of US28-WT and US28-R129A (A), US28-WT and empty vector (B), and US28-R129A and empty vector (C). In each case, the relative abundance of human proteins MNDA and IFI16 is marked with a black arrowhead or arrow. (D) Analysis of the top 40 downregulated proteins identified in panel A. After filtering for changes with a *q* value of <0.001, the gene names were entered in the Interferome database. Proteins that are induced by type I and/or type II interferon are depicted in the Venn diagram. Proteins that we identified that are not interferon inducible are listed below (under “Neither”). (E) Lysates from THP-1 cells expressing empty vector (EV), US28-WT, and US28-R129A were assessed by Western blotting for phospho-STAT1 (Tyr701), total STAT1, and beta-actin (loading control). (F and G) Quantification of STAT1 and phospho-STAT1 band intensity from two Western blots from two independent samples of transduced THP-1 cells. (F) Quantification of the indicated protein levels normalized to actin. (G) Quantification of phospho-STAT1 levels relative to total STAT1.

US28-mediated signaling is critical to latency: US28-deleted viruses, or the loss of G-protein coupling by the US28 mutant R129A, results in lytic infection of undifferentiated myeloid cells (19, 21, 23, 25). Similarly, infection of US28-WT-expressing THP-1 cells with US28 deletion viruses leads to complementation and the establishment of latency; both the US28-R129A- and empty-vector-expressing cell lines fail to establish latent infection under these conditions (21). Therefore, to understand how US28-WT alters the host cell environment to support latency, we analyzed the total proteomes of myelomonocytic THP-1 cell lines which express either US28-WT (sequence derived from the VHL/E strain of HCMV), signaling mutant US28-R129A, or the empty vector (EV) control (Fig. 1A to C; see also Data Set S1 in the supplemental material).

10.1128/mBio.02574-19.5 DATA SET S1 US28 proteome in THP-1 cells. (Tab 1, or Data tab) THP-1 cells expressing empty vector, US28-WT, and US28-R129A were subjected to total cell proteomic analysis using a TMT labeling approach as described in Materials and Methods. This file lists all genes identified in this proteomic screen, including their UniProt accession number, HUGO gene symbol, fold changes in abundance between cell lines, and *q* values of statistical significance. (Tab 2, or Interferome Top 40 Downreg tab) Gene names, fold changes, and *q* values of the top 40 most downregulated genes (US28-WT versus US28-R129A) are presented, along with whether they are included in the Interferome database as being type I or type II interferon inducible (marked “y” for yes). (Tab 3, or Interferome Zero-Change 40 tab) Gene names, fold changes, and *q* values of the genes with a fold change value of zero (US28-WT versus US28-R129A) are presented, along with whether they are included in the Interferome database as being type I or type II interferon inducible (marked “y” for yes). Download Data Set S1, XLSX file, 1.5 MB. Copyright © 2019 Elder et al. 2019 Elder et al. This content is distributed under the terms of the Creative Commons Attribution 4.0 International license.

By using these three cell lines, we were able to identify host cell proteins modulated by US28 expression in a G-protein signaling-dependent and -independent manner. Changes in host protein abundance common between cells expressing US28-WT and US28-R129A compared to cells expressing empty vector (Fig. 1B and C) represent signaling-independent changes, and these include CD44 and CD82 proteins, which are each downregulated by both sets of US28-expressing cells.

While we do not rule out the possibility that signaling independent changes in myeloid cells driven by US28 may be important for HCMV latency, G-protein-dependent signaling is absolutely required for latency, and therefore, we were particularly interested in the direct comparison of host protein abundances in THP-1 cells expressing US28-WT and US28-R129A (Fig. 1A). This comparison reveals 42 host proteins whose expression is twofold or more increased or decreased by US28-WT, and our analyses focused on these signaling-dependent changes.

One remarkable feature of many of the most downregulated proteins in Fig. 1A is that they are interferon inducible (Fig. 1D; see also Fig. S1A in the supplemental material). According to the Interferome database (v2.01) (www.interferome.org) (42), two-thirds (27/40) of the most downregulated proteins (fold change 1.86-fold or higher) we identified are type I or type II interferon inducible (Fig. 1D). In contrast, of the 40 proteins which showed no changes (fold change = 0) in abundance between US28-WT and US28-R129A, 12/40 (30%) were included in the Interferome database (Data Set S1). Importantly, overexpression of the multipass membrane US28 proteins did not lead to induction of the unfolded protein response or other endoplasmic-stress-related genes (Fig. S1B), suggesting that the changes identified in the screen are not general effects of protein overexpression.

10.1128/mBio.02574-19.1 FIG S1 US28 expression induces IFN-inducible genes, but not endoplasmic reticulum (ER) stress-related genes. (A) Changes in interferon-inducible genes identified in Fig. 1D, and other canonical ISGs, in US28-WT with respect to US28-R129A. Green bars indicate changes with a *q* value of <0.001. (D) Heat map of the changes in canonical ER stress-related genes induced by US28-WT or US28-R129A expression as per the proteomic screens in Fig. 1A to C. HUGO gene symbols are listed followed by a common gene name, if applicable. An outgroup of genes that are regulated by US28 (IFI16, MNDA, FLT3) is included for comparison. Download FIG S1, JPG file, 0.6 MB. Copyright © 2019 Elder et al. 2019 Elder et al. This content is distributed under the terms of the Creative Commons Attribution 4.0 International license.

Since STAT1 phosphorylation is common to both the type I and type II interferon signaling pathways (43), we examined total STAT1 and phosphorylated STAT1 in US28-WT cells. We found that US28-WT cells had lower overall levels of STAT1 and phosphorylated STAT1 compared to cells transduced with US28-R129A or empty vector control (Fig. 1E and F). Furthermore, when correcting for total levels of STAT1, we found that US28-WT cells had lower relative levels of phosphorylated STAT1 compared with US28-R129A (Fig. 1E and G). This could help explain why US28-WT downregulated many interferon-inducible genes in our proteomic screen.

### IFI16, MNDA, and HLA-DR are all downregulated by US28.

**FIG 2:**
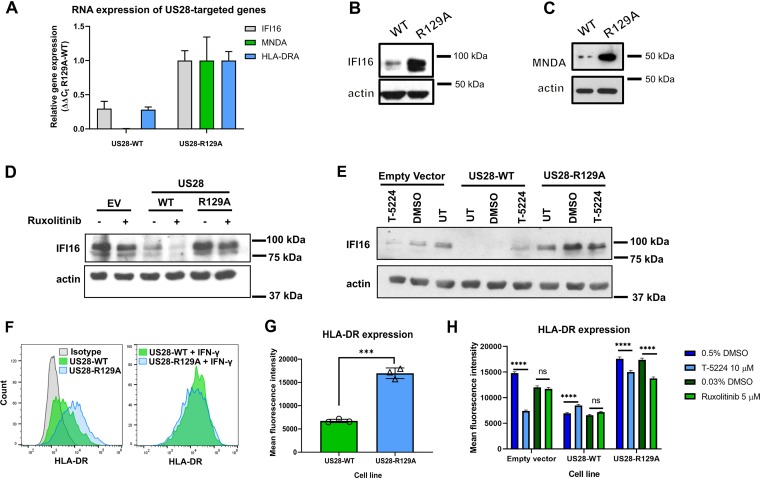
US28-expressing cell lines downregulate IFI16, MNDA, and HLA-DR. (A) Relative RNA expression of IFI16, MNDA, and HLA-DR in US28-expressing THP-1 cells. The levels of IFI16, MNDA, and HLA-DRA were normalized to the levels of TBP and then to US28-R129A using the ΔΔ*C_t_* method. (B and C) Lysates from cells expressing US28-WT and US28-R129A were analyzed by Western blotting for IFI16 (B) and MNDA (C) expression; actin is shown as a loading control. (D) Cells expressing empty vector (EV), US28-WT, and US28-R129A were treated with 5 μM ruxolitinib (+) or with an equivalent concentration of DMSO (−), for 48 h, before analysis for IFI16 expression by Western blotting, using actin as a loading control. (E) As in panel D, except that cells were treated with T-5224 at 10 μM, DMSO, or left untreated (UT). (F) US28-expressing cells were maintained in culture media only (left panel) or treated with 1 ng/ml of IFN-γ (right panel) for 24 h before staining for cell surface HLA-DR by flow cytometry. Staining was performed in triplicate for untreated cells, and the mean of these is experiments is presented in panel G as mean fluorescence intensity with standard deviation. Statistical analysis was performed by one-way analysis of variance (ANOVA), and statistical significance is indicated by a bar and asterisks as follows: ***, *P* < 0.001. (H) As in panels D and E, except cells were analyzed for cell surface HLA-DR by flow cytometry, and results are presented as mean fluorescence intensity with 95% confidence intervals. Statistical analysis was performed by two-way ANOVA using Bonferroni’s multiple-comparison test, and statistical significance is indicated as follows: ns, not significant (*P* > 0.01); ****, *P* < 0.0001.

Several interferon-inducible proteins showing decreased expression were of interest as potentially important targets for US28 during HCMV latency. These proteins included MNDA (nine unique peptides; 5.0-fold downregulated compared to US28-R129A), IFI16 (four unique peptides; 2.4-fold downregulated compared to US28-R129A), and components of the MHC class II HLA-DR complex (three to six unique peptides; between 1.9- and 2.2-fold downregulated compared to US28-R129A). We began by confirming US28-WT-mediated downregulation of these proteins in independently transduced US28-expressing THP-1 cells. After generating these fresh US28-expressing cell lines, we checked expression levels of US28-WT or US28-R129A by reverse transcription-quantitative PCR (RT-qPCR) (Fig. S2A) and Western blotting (Fig. S2B and S2C). RT-qPCR confirmed that IFI16, MNDA, and HLA-DRA transcripts are all downregulated in US28-WT-expressing cells compared to those expressing the signaling mutant R129A (Fig. 2A). Subsequently, we confirmed this US28-WT-mediated downregulation of IFI16 and MNDA at the protein level by Western blotting (Fig. 2B and C and Fig. S2D, S2E, and S2F) or by flow cytometry for cell surface HLA-DR (Fig. 2F and G), although both US28-WT and US28-R129A cells responded similarly to gamma interferon (IFN-γ) stimulation, by upregulating HLA-DR to similar levels (Fig. 2F). This suggests that, while US28 is able to downregulate constitutive HLA-DR expression, it is unable to prevent its induction by IFN-γ. Furthermore, this downregulation was not due to strain-specific effects of US28, as US28-WT also downregulated HLA-DR when the US28 sequence from the TB40/E strain of HCMV was used to transduce THP-1 cells (Fig. S3A to D).

10.1128/mBio.02574-19.2 FIG S2 US28-expressing cell lines downregulate IFI16, MNDA, and HLA-DR. (A) Empty-vector-, US28-WT-, and US28-R129A-expressing THP-1 cells were regenerated in independent transductions using the same expression vectors used for the proteomic screen (Fig. 1). US28 expression was validated by RT-qPCR, with US28 RNA normalized to TATA box binding protein (TBP) and presented as 2^-ΔCt^. (B) Cells from panel A were lysed and subjected to Western blotting for US28, and actin was used as a loading control. (C) Quantification of three Western blots for US28 expression. (C and D) Lysates prepared from cells in panel A were analyzed by Western blotting for IFI16 (C) and MNDA (D) expression; actin is shown as a loading control. Note that panel E is from the same membrane as Fig. 1C. (F) Quantification of five and four independent Western blots for IFI16 and MNDA, respectively. (G) Cells from panel A were treated with ruxolitinib as in Fig. 2D or left untreated. Lysates from these cells were analyzed by Western blotting for phosphorylated STAT1, total STAT1, or actin as a loading control. Download FIG S2, JPG file, 0.7 MB. Copyright © 2019 Elder et al. 2019 Elder et al. This content is distributed under the terms of the Creative Commons Attribution 4.0 International license.

10.1128/mBio.02574-19.3 FIG S3 Strain-dependent differences in US28 do not affect downregulation of interferon-inducible genes. (A) Sequences encoding US28 from the indicated HCMV strains or plasmids were aligned using Clustal Omega. (B) Retroviral plasmids encoding US28-WT (from TB40/E) or R129A, each with a C-terminal 3XFLAG tag, and an eGFP marker, were used to transduce THP-1 cells. They were then subjected to immunofluorescence staining for the 3XFLAG tag. (C and D) Cells from panel B were stained for cell surface HLA-DR by flow cytometry. (D) Mean fluorescence intensity of the US28-WT and US28-R129A cell lines. Statistical analysis was performed by Student’s t test. Statistical significance is indicated as follows: **, *P* < 0.01. Download FIG S3, JPG file, 2.2 MB. Copyright © 2019 Elder et al. 2019 Elder et al. This content is distributed under the terms of the Creative Commons Attribution 4.0 International license.

Since US28 attenuates c-*fos* signaling (25) and STAT1 phosphorylation (Fig. 1E to G), and both HLA-DR and IFI16 are *fos* and STAT1-responsive genes (44–48), we hypothesized that one or both of these mechanisms is responsible for US28-mediated downregulation of IFI16 and HLA-DR. To test this, we treated THP-1 cells expressing empty vector, US28-WT, and US28-R129A with the Janus kinase inhibitor Ruxolitinib or the c-*fos* inhibitor T-5224. Ruxolitinib partially downregulated IFI16 expression in all three cell types (Fig. 2D) but downregulated HLA-DR only in the US28-R129A-expressing cell line (Fig. 2H), despite a complete block in STAT1 phosphorylation (Fig. S2G). The c-*fos* inhibitor reduced IFI16 and HLA-DR expression in comparison with dimethyl sulfoxide (DMSO) controls in empty-vector -and R129A cell lines, and in the case of empty vector, this drop in expression was almost down to the level in untreated US28-WT cells (Fig. 2E and H). Taken together, we think it likely that both c-*fos* and STAT1 attenuation are important for US28-mediated downregulation of IFI16 and HLA-DR. Interestingly, the c-*fos* inhibitor actually increased IFI16 expression in US28-WT-expressing cells. We think that this could be due to a basal level of c-*fos* being required for the expression of a host gene, as yet unidentified, that is needed by US28-WT to attenuate multiple signaling pathways; one candidate gene for this is the AP-1-inducible phosphatase DUSP1 (49) which will require further investigation.

### Latent infection of monocytes is associated with the downregulation of IFI16, MNDA, and HLA-DR.

**FIG 3:**
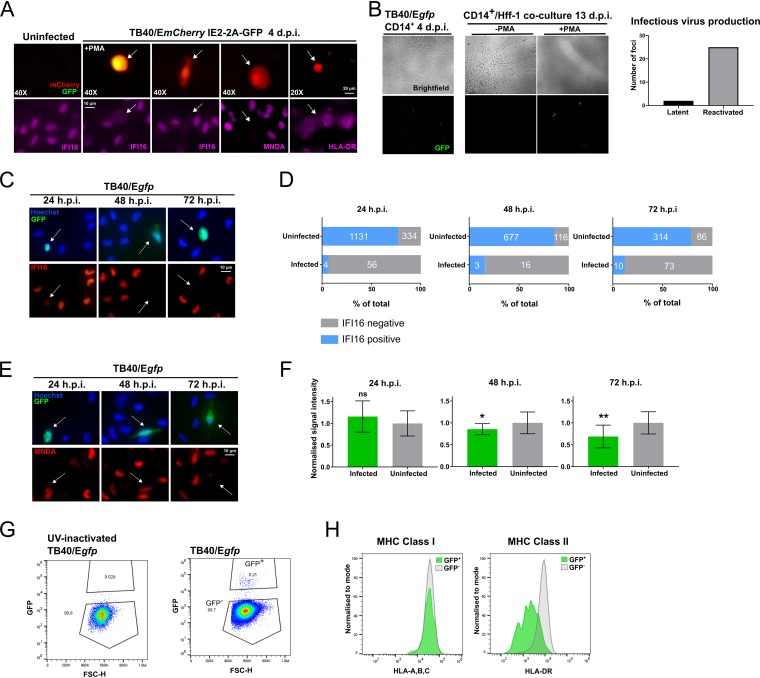
IFI16, MNDA, and HLA-DR are downregulated in latently infected CD14^+^ monocytes. Primary CD14^+^ monocytes were isolated from peripheral blood or apheresis cones as described in Materials and Methods. These cells were then infected using bacterial artificial chromosome (BAC)-derived strains of TB40/E. (A) CD14^+^ monocytes latently infected with TB40/E SV40-mCherry IE2-2A-GFP stained by immunofluorescence for IFI16, MNDA, or HLA-DR as indicated at 4 days postinfection (d.p.i.) and imaged by wide-field fluorescence microscopy. The top left images show uninfected monocytes. The second from the left images show monocytes that were treated with PMA (+PMA) (to permit lytic infection). mCherry (red) serves as a marker for infection, and GFP (green) denotes expression from the IE2-2A-GFP cassette. The remaining images show monocytes cultured in the absence of PMA. The absence of green fluorescence results from suppressed expression of the IE2-2A-GFP cassette and scored as IE negative. The magnification is indicated (40X or 20X). White arrows indicate corresponding cells in the top and bottom panels. (B) Validation of experimental latency using TB40/E*gfp* virus. CD14^+^ monocytes were infected and allowed to establish latency for 4 days (left panel, ×10 magnification). Citrate wash buffer was used to remove externally bound virions. These latently infected cells were cultured in the presence (+) or absence (−) of PMA for 3 days, and at 7 d.p.i., Hff-1 cells were added to the culture to demonstrate production of infectious virions. Transfer of virus to Hff-1 cells was monitored by fluorescence microscopy up to 13 d.p.i., and infected Hff-1 foci were counted and summed across the experiment (three wells of CD14^+^ monocytes per condition, graphed). (C) CD14^+^ monocytes infected with TB40/E*gfp* stained by immunofluorescence for IFI16 at 24, 48, and 72 h.p.i. and imaged as before using ×60 magnification. (D) Quantification of IFI16-positive and -negative monocytes in the uninfected and infected populations from two donors per time point. Raw numbers of cells are indicated in white text. Fisher’s exact test indicates a statistically significant difference between uninfected and infected populations for each time point (*P* < 0.0001). (E) CD14^+^ monocytes infected with TB40/E*gfp* were stained by immunofluorescence for MNDA at the indicated times and imaged as before using ×60 magnification. (F) Quantification of the signal intensity from infected monocytes at the indicated time points (*n* = 9, 7, and 10 for the 24-, 48-, and 72-h.p.i. graphs, respectively). MNDA signal intensity in each nucleus was normalized to the average of uninfected monocytes from each field of view. A *t* test with Welch’s correction was used to determine statistical significance. Statistical significance is indicated as follows: ns, not significant; *, *P* < 0.05; **, *P* < 0.01. (G) CD14^+^ monocytes infected with TB40/E*gfp* (with or without UV inactivation) were analyzed for HLA-ABC and HLA-DR expression at 3 d.p.i. by flow cytometry. The gating strategy for identifying infected cells (GFP^+^) is shown. FSC, forward scatter. (H) Histogram showing HLA-ABC and HLA-DR staining in HCMV-uninfected GFP-negative (gray) monocytes, and latently infected GFP-positive (green) monocytes.

Having confirmed key observations from our proteomic data in transduced THP-1 cells overexpressing US28 in isolation, we then sought to determine whether IFI16, MNDA, and HLA-DR are also downregulated in an experimental model of latency in *ex vivo* primary CD14^+^ monocytes where latency-associated expression of US28 is well established. To do this, we infected CD14^+^ monocytes with TB40/E-BAC4 strains of HCMV engineered to express either green fluorescent protein (GFP) or mCherry as markers for latent or lytic infection. First, we analyzed CD14^+^ monocytes infected with TB40/E SV40 mCherry/IE2-2A-GFP. This virus drives constitutive mCherry expression in all infected cells via the simian virus 40 (SV40) promoter, but GFP expression is restricted to lytically infected cells as a result of IE2 expression, which is linked to GFP by the self-cleaving peptide 2A. Therefore, we were able to distinguish IE2-positive (lytic) cells from IE2-negative cells (one hallmark of latency) among infected, mCherry-positive cells. At 4 days postinfection (d.p.i.), we fixed and immunostained the monocytes for our cellular proteins of interest in mCherry-positive, IE2-2A-GFP-negative cells (Fig. 3A). For a control, we also differentiated monocytes with phorbol 12-myristate 12-acetate (PMA), which drives IE2-2A-GFP expression through differentiation-dependent reactivation. We found that IFI16, MNDA, and HLA-DR were all downregulated in latently infected, mCherry-positive but IE2-negative, CD14^+^ monocytes. Importantly, this comparison held true when comparing infected monocytes with uninfected monocytes that had not had contact with viral particles (Fig. 3A and Fig. S4A).

10.1128/mBio.02574-19.4 FIG S4 Downregulation of IFI16, MNDA, and HLA-DR is not simply a bystander effect of contact with viral particles. (A) CD14^+^ monocytes were left uninfected or infected with HCMV for 24 h before fixing and staining for the indicated proteins, and imaging as before. (B) The sequence encoding US28 from VHL/E was cloned into the lentiviral plasmid pUbEm (US28-UbEm), and this or empty UbEm plasmid was used to transduce THP-1 cells, which were subsequently cell sorted for Emerald expression. (C) US28 expression was validated in the cells from panel B by RT-qPCR. The level of US28 RNA was normalized to the level of cellular TBP and presented as 2^-ΔCt^. Download FIG S4, JPG file, 1.0 MB. Copyright © 2019 Elder et al. 2019 Elder et al. This content is distributed under the terms of the Creative Commons Attribution 4.0 International license.

We then sought to look at expression of these proteins at earlier time points. US28 is a virion-associated protein (19), and incoming US28 is reported to have rapid effects on host cells (25). We speculated that the downregulation of IFI16, MNDA, and HLA-DR might occur early during the establishment of latency. For these experiments, we used TB40/E*gfp* which marks infected cells with GFP expression via the SV40 promoter and confirmed the establishment of latency in this system by coculture of monocytes with fibroblasts either with or without PMA-induced reactivation (Fig. 3B). In our latency system, we found a stark and specific loss of IFI16 in infected monocytes from 24 h postinfection (h.p.i.) (Fig. 3C), a phenotype maintained at 48 and 72 h.p.i. (Fig. 3C) as measured by immunofluorescence. We quantified these observations in several fields of view for each of these three time points and performed contingency analyses (Fisher’s exact test), which confirmed specific loss of IFI16 in latently infected cells (Fig. 3D). Loss of IFI16 was observed in *ex vivo* infected monocytes at these time points in a total of four separate donors with TB40/E*gfp* virus.

We found a partial downregulation of MNDA by 72 h.p.i. (Fig. 3E and F), with a very small downregulation at 48 h.p.i. and no effect at 24 h.p.i., suggesting that modulation of MNDA is delayed compared with a fellow PYHIN family member, IFI16. We also observed that HLA-DR, but not corresponding MHC class I HLA-A,B,C, was downregulated at 72 h.p.i. specifically in GFP-positive, latently infected monocytes (Fig. 3G and H). Therefore, IFI16, MNDA, and HLA-DR are indeed downregulated at early times during the establishment of latency, with IFI16 showing downregulation within 24 h of infection.

### The downregulation of IFI16 is dependent on viral US28.

**FIG 4:**
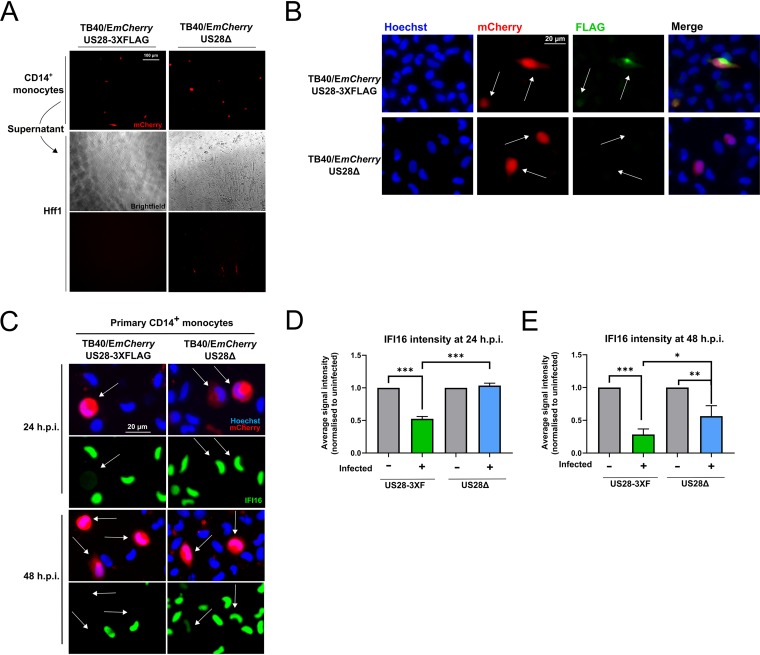
IFI16 is rapidly downregulated in a US28-dependent manner during latent infection. CD14^+^ monocytes were infected with either US28 WT TB40/E*mCherry*-US28-3XFLAG HCMV or ΔUS28. (A) Validation of the latent and lytic phenotypes associated with US28-3xF and ΔUS28 monocyte infections, respectively. At 7 d.p.i., supernatant from infected CD14^+^ cells (top panel) were transferred to Hff1 cells (middle bright-field and bottom mCherry panels), and formation of plaques was monitored and imaged at ×20 magnification. (B) Detection of US28-3XFLAG during the establishment of latency in CD14^+^ monocytes. At 2 d.p.i., US28-3xF- or ΔUS28-infected CD14^+^ monocytes were fixed and stained by immunofluorescence for US28-3XFLAG using an anti-FLAG antibody and imaged at ×40 magnification. (C) US28-3XF- and ΔUS28-infected monocytes were stained by immunofluorescence for IFI16 at the indicated times and imaged using ×40 magnification. White arrows indicate corresponding cells. (D and E) IFI16 signal intensity in each nucleus was normalized to the average of the uninfected cells in a field of view. The results of three fields of view were then averaged to derive the resulting average signal intensities for each subpopulation of monocytes at the indicated time points infected with US28-3xF or ΔUS28 HCMV. Statistical significance was determined using one-way ANOVA and is indicated by asterisks as follows: ***, *P* < 0.001; **, *P* < 0.01; *, *P* < 0.05.

Having confirmed that IFI16 is downregulated very early during latent infection of monocytes, we then sought to establish whether this effect is dependent on US28. We predicted this would be the case because of the results of our US28 proteomic screen and the established functionality of incoming virion-associated US28 (25). We infected monocytes with either the US28 WTTB40/E*mCherry*-US28-3XFLAG HCMV (US38-3XF) or the corresponding US28 deletion virus TB40/E*mCherry*-US28Δ (ΔUS28). These viruses establish latent and lytic infections, respectively, in CD34^+^ progenitor cells, Kasumi-3 cells, and THP-1 cells (19, 25), and we confirmed that these phenotypes are also maintained in primary CD14^+^ monocytes by supernatant transfer to permissive fibroblasts (Fig. 4A). We were also able to detect US28 protein during the establishment of latency in monocytes by immunostaining for the FLAG epitope tag on the C terminus of US28 (Fig. 4B).

To determine whether US28 specifically downregulates IFI16 in the context of infection, we compared the expression of this cellular protein in monocytes infected with US28-3XF or ΔUS28. Consistent with Fig. 3B, we found that monocytes infected with the US28-3xF virus showed downregulation of IFI16 at 24 and 48 h.p.i., while monocytes infected with ΔUS28 displayed robust IFI16 expression at 24 h.p.i. (Fig. 4C and D) and only partial downregulation at 48 h.p.i. (Fig. 4C and E). These data demonstrate that the early downregulation of IFI16 in CD14^+^ monocytes is dependent on US28.

### IFI16 is downregulated by US28 only in undifferentiated myeloid cells.

**FIG 5:**
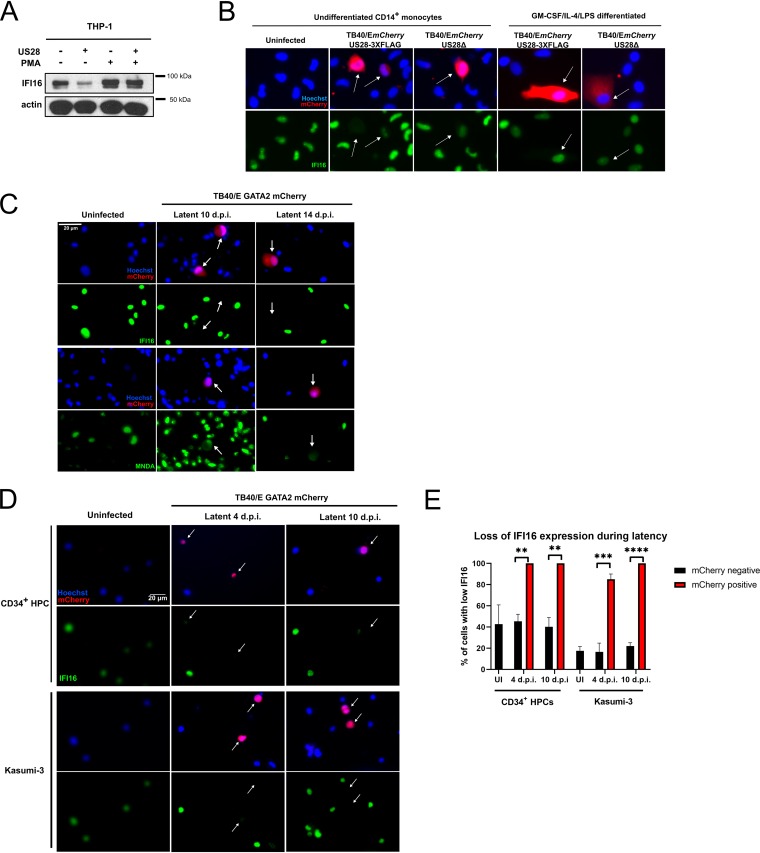
IFI16 downregulation is maintained during long-term latency of undifferentiated monocytes and CD34^+^ progenitor cells. (A) US28-expressing and empty-vector-expressing THP-1 cells were either left untreated or treated with PMA for 48 h before cell lysates were harvested. These lysates were then subjected to Western blotting for IFI16 and actin as a loading control, with molecular weight markers (in kilodaltons) annotated. (B) At 48 h.p.i., either undifferentiated CD14^+^ monocytes, or monocytes predifferentiated for 7 days with GM-CSF/IL-4/LPS were fixed and stained for IFI16 and imaged as before at ×40 magnification. White arrows indicate corresponding infected cells. (C) CD14^+^ monocytes were infected with HCMV GATA2mCherry or left uninfected. At the indicated times, cells were fixed and stained for IFI16 or MNDA and imaged as before. (D) Primary CD34^+^ hematopoietic progenitor cells from two donors, or Kasumi-3 cells, were infected with HCMV GATA2mCherry or left uninfected. At the indicated times, cells were fixed and stained for IFI16 and imaged as before. (E) Quantification of at least three fields of view from panel D, presented as the proportion of cells with low IFI16 expression in the infected, mCherry-positive and uninfected, mCherry-negative populations. UI, uninfected. Statistical analysis was performed by Fisher’s exact test on the total number of cells in each category. Statistical significance is indicated by asterisks as follows: ****, *P* < 0.0001; ***, *P* < 0.001; **, *P* < 0.01.

We previously showed that US28 modulates cellular signaling pathways in undifferentiated, but not differentiated, THP-1 cells (21). We were therefore curious as to whether the effects on IFI16 expression were dependent on cellular differentiation status. This is significant because differentiated THP-1 cells and mature dendritic cells are permissive for HCMV lytic infection. To analyze whether these effects are differentiation dependent, we transduced THP-1 cells with a lentiviral vector that coexpresses US28 and the fluorescent protein Emerald (US28-UbEm), or coexpresses enhanced GFP (eGFP) and Emerald (empty UbEm) as a control. For each population, we sorted the Emerald-positive THP-1 cells by fluorescence-activated cell sorting (FACS) (Fig. S4B) and validated US28 expression by RT-qPCR (Fig. S4C). We treated half of these cells with PMA in order to induce cellular differentiation. We found that undifferentiated US28-expressing THP-1 cells downregulated IFI16, but PMA-differentiated cells did not downregulate IFI16 (Fig. 5A), suggesting that only latency-associated expression of US28 attenuates IFI16 expression.

We also analyzed the effect of cellular differentiation on IFI16 expression following infection in mature dendritic cells derived by treating *ex vivo* CD14^+^ monocytes with granulocyte-macrophage colony-stimulating factor (GM-CSF)/interleukin-4 (IL-4)/lipopolysaccharide (LPS). Again, we found that undifferentiated infected CD14^+^ monocytes downregulate IFI16 in a US28-dependent manner at 48 h.p.i., while infected mature dendritic cells do not downregulate IFI16 with WT or ΔUS28 HCMV (Fig. 5B). Taken together, our results indicate that US28 rapidly downregulates IFI16 during latent infection of monocytes, but not during lytic infection of mature dendritic cells.

### Low levels of IFI16 are maintained during long-term latency in monocytes and CD34^+^ progenitor cells.

We next assessed whether downregulation of IFI16 and MNDA occurs during long-term maintenance of latency; long-term downregulation of HLA-DR is already known to be important for latent carriage of HCMV (41). We infected monocytes with HCMV that drives mCherry from the GATA2 promoter and maintains this marker for far longer during latency than SV40 promoter-driven tags (50). At 10 and 14 d.p.i., IFI16 remained absent and MNDA remained partially downregulated in infected cells (Fig. 5C). We then repeated this analysis in primary CD34^+^ hematopoietic progenitor cells (HPCs), a site of long-term *in vivo* latent carriage, as well as the Kasumi-3 cell line, an experimental model for HCMV latency (51). Consistent with our observations in monocytes and transcriptome sequencing (RNAseq) experiments in cord blood-derived CD34^+^ cells (52), IFI16 levels were low or absent in almost all infected cells imaged at 4 and 10 days postinfection (Fig. 5D and E). Thus, it seems likely that downregulation of IFI16 is a conserved process in cellular sites of HCMV latency.

### IFI16 activates IE gene expression via NF-κB in myeloid cells.

**FIG 6:**
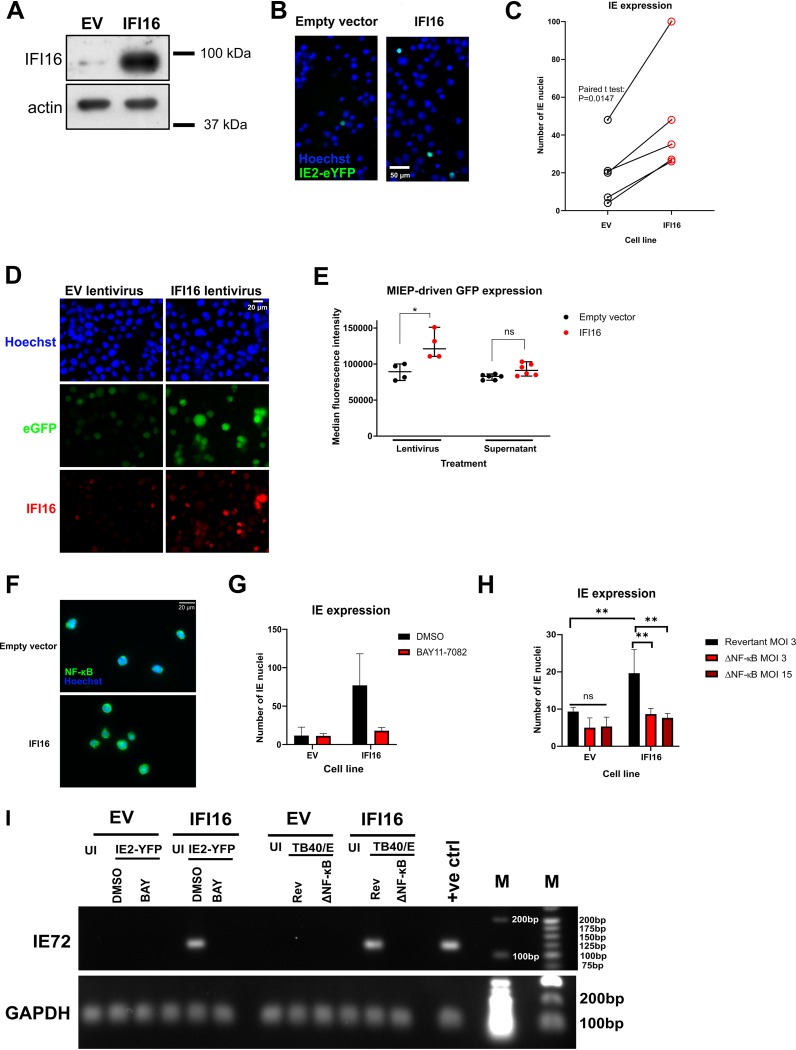
Overexpression of IFI16 in monocytic cells leads to MIEP activation and IE gene expression via NF-κB. (A) THP-1 cells were transduced with empty vector (EV) or IFI16-overexpressing lentiviruses and after blasticidin selection, IFI16 overexpression was confirmed by Western blotting. Actin is shown as a loading control. (B and C) Cells expressing empty vector or overexpressing IFI16 (from panel A) were infected with TB40/E IE2-eYFP (enhanced YFP) virus, and IE2-YFP-positive nuclei were imaged and counted by fluorescence microscopy. (C) Cumulative results from five paired experiments, which were analyzed by paired two-tailed Student’s *t* test; *, *P* < 0.05. (D) EV and IFI16 lentivirus concentration was determined by p24 ELISA (data not shown) and 15 ng p24 equivalents of each lentivirus was used to transduce MIEP-eGFP THP-1 cells. Cells were maintained for 2 weeks in culture, and IFI16 overexpression was validated by immunofluorescence. (E, left-hand comparison) Cells shown in panel D were assessed for eGFP fluorescence by flow cytometry. Right-hand comparison, nontransduced MIEP-eGFP-expressing cells were incubated with supernatants from cells described above in the legend for panel A for 2 days. eGFP expression was quantified by flow cytometry. A statistical comparison of the median fluorescence intensity was performed using two-tailed Mann-Whitney test, and statistical significance is indicated as follows; ns, not significant, *, *P* < 0.05. (F) Empty-vector- or IFI16-overexpressing cells were fixed and stained for NF-κB, with Hoechst as a nuclear stain, at ×40 magnification to assess the levels of nuclear NF-κB. (G) Empty-vector- or IFI16-overexpressing cells were infected with TB40/E IE2-eYFP virus in the presence of the IKKα inhibitor BAY11-7082, which inhibits the NF-κB pathway, or DMSO as a control. IE2-eYFP-positive nuclei were imaged and counted by fluorescence microscopy at 48 h postinfection. (H) Empty-vector- or IFI16-overexpressing cells were infected with a revertant WT-like TB40/E at an MOI of 3 or with TB40/E with NF-κB binding sites deleted from the MIEP (ΔNF-κB) at an MOI of 3 or 15. At 48 h.p.i., cells were fixed and stained for IE, and the numbers of IE-positive nuclei were counted. The graph shows the results of three experiments and statistical analysis by two-way ANOVA using Sidak’s multiple-comparison test. Statistical significance is indicated as follows: **, *P* < 0.01; ns, not significant (*P* > 0.05). (I) Empty-vector- or IFI16-overexpressing cells were infected as in panels F and H at an MOI of 3, but cells were instead analyzed for IE72 expression by RT-qPCR. PCR products were then run on a 2% (IE72) or 1.2% (GAPDH) agarose gel. UI refers to uninfected cells, DMSO is the solvent control, BAY refers to BAY11-7082, Rev refers to the revertant TB40/E, and ΔNF-κB refers to the NF-κB binding site mutant virus. The positive control (+ve ctrl) was HCMV-infected PMA-differentiated monocytes. Molecular weight markers (M) are annotated (in base pairs).

Having established that US28 downregulates IFI16 early during the establishment of latency, we wanted to address why this may be beneficial to the virus for latent infection. One function of IFI16 is the sensing of viral DNA and subsequent induction of type I interferon or interleukin-1beta (27, 52). While we do not currently rule out a potential role for IFI16-mediated sensing of HCMV during latency, we were more struck by the previous work identifying IFI16 as a modulator of host and viral transcription (32–34, 36, 37, 53–58). In particular, IFI16 is capable of activating the MIEP and driving IE gene expression within the first 6 h of lytic infection of fibroblasts (32, 34), though at later times IFI16 blocks early and late gene expression (34, 37). Given that a hallmark of HCMV latency is the suppression of IE gene expression (59), we hypothesized that high levels of IFI16 might drive MIEP activity and IE gene expression in undifferentiated myeloid lineage cells. To address this, we transduced and selected THP-1 cells with control empty-vector- or IFI16-overexpressing lentiviruses to generate control and IFI16-overexpressing cell lines. We validated IFI16 overexpression by Western blotting (Fig. 6A) and then infected these cell lines with recombinant HCMV carrying an IE2-YFP (yellow fluorescent protein) cassette to allow us to identify cells that express IE2 (60). Undifferentiated THP-1 cells are an established model for a number of aspects of HCMV latency (61), including the significant repression of IE2 expression (61). When we infected control and IFI16-overexpressing THP-1 cells with HCMV in five paired experiments, we found a consistent increase in the number of IE-positive cells in IFI16-overexpressing THP-1 cells (Fig. 6B and C), suggesting IFI16 overexpression drives IE protein production in cells that would otherwise repress this viral protein.

Prior work has identified that IFI16 could activate IE gene expression in a UL83-dependent manner during lytic infection (32, 37), but since this tegument protein does not enter the nucleus in the CD34^+^ progenitor cell model of HCMV latency (62), we hypothesized that IFI16 could activate the MIEP without additional virion components. To test this hypothesis, we utilized a THP-1 MIEP reporter system; THP-1 cells in which an integrated 1,151-bp region of the MIEP drives the expression of eGFP (63). In these undifferentiated THP-1 cells, the MIEP is epigenetically repressed unless stimulated (for example by differentiation) (63). We treated these MIEP-eGFP THP-1 cells with control lentiviruses or lentiviruses which drive the overexpression of IFI16, ensuring equivalent lentivirus infection of reporter cells by a p24 enzyme-linked immunosorbent assay (ELISA). These cultures were maintained for 2 weeks, after which we validated IFI16 expression by immunofluorescence (Fig. 6D) and then analyzed eGFP expression by flow cytometry (Fig. 6E). We found that the IFI16-overexpressing cells had increased eGFP expression compared with controls (Fig. 6D and E), suggesting that IFI16 overexpressed in isolation and in the absence of additional HCMV components drives MIEP activity. Furthermore, culturing THP-1 MIEP-eGFP reporter cells with supernatants from the empty-vector- or IFI16-overexpressing cell lines in Fig. 6A resulted in no significant MIEP activity, suggesting that the effect is mediated intracellularly, and not by a secreted factor (Fig. 6E).

IFI16 activates NF-κB signaling in a number of contexts (53, 64), and our previous work indicates that US28-mediated attenuation of NF-κB signaling is important for the establishment of latency (21). Therefore, we hypothesized that IFI16 activates the MIEP via NF-κB. We found increased nuclear NF-κB localization in IFI16-overexpressing cells (Fig. 6F), and by using the NF-κB pathway inhibitor, BAY11-7082, we were able to ameliorate the effect of IFI16 overexpression on IE (Fig. 6G and I), suggesting that NF-κB plays an important role in this pathway. Finally, we infected IFI16-overeexpressing cells with HCMV that lacks NF-κB sites within the MIEP (65) to check whether IFI16 exerts its effects via direct binding of NF-κB to the MIEP. In this case, IFI16 overexpression failed to induce IE gene expression, unlike IFI16 cells infected with the revertant strain (Fig. 6H and I). Taken together, our data are consistent with the view that IFI16 activates IE gene expression in early myeloid lineage cells by allowing NF-κB to bind at the MIEP.

## DISCUSSION

The viral GPCR US28 is expressed during both lytic and latent infection of HCMV. While US28 is dispensable for lytic replication *in vitro* (66, 67), it is essential for the establishment and maintenance of HCMV latency in early myeloid lineage cells (19, 21, 23, 25). This is attributable, in part, to the ability of US28 to suppress the major immediate early promoter; a US28 function specific for undifferentiated myeloid cells (18, 21, 23, 25).

We hypothesized that this ability of US28 to so profoundly regulate viral IE gene expression in undifferentiated myeloid cells was likely via US28-mediated modulation of host protein abundance, and therefore, we wanted to determine whether such US28-driven changes could be important for the establishment or maintenance of HCMV latency. While previous work has used targeted arrays to assess US28-mediated effects on myeloid cells (21, 23, 25), here we have used an unbiased proteomic screen to understand how US28 reprograms host cells in order to support latent infection. Our screen compared host protein abundance in control THP-1 cells or THP-1 cells which express either WT-US28 or the US28 signaling mutant US28-R129A. As such, we could assess the signaling-dependent and signaling-independent effects of US28. We then chose to focus on signaling-dependent changes because G-protein coupling via the residue R129A is essential for experimental latency (21, 25). However, we predict that some of the signaling-independent changes driven by US28 could also be important for HCMV latency, since these changes included alterations in several cell surface molecules such as costimulatory molecule CD82, adhesion molecule CD44, and in receptor tyrosine kinase FLT3. The latter two cellular factors are implicated in myeloid cell differentiation, which is intimately linked with HCMV latency and reactivation (18, 59, 68–70). As such, modulating these cell surface molecules could help to control interactions with immune effectors and cellular differentiation-linked reactivation.

By looking at changes in host protein abundance between US28-WT- and US28-R129A-expressing THP-1 cells, we found a number of significant changes in the host proteome which likely result specifically from US28 signaling. Interestingly, we found US28-WT downregulated a large number of interferon-inducible proteins, including canonical interferon-stimulated genes (ISGs) like OAS2 and IFITM3, as well as MNDA, IFI16, and several HLA-DR components. We found that the levels of both total STAT1 and phosphorylated STAT1 were reduced in US28-WT-expressing cells, a mechanism that may act in synergy with the US28-mediated attenuation of c-*fos* to downregulate interferon-inducible genes (25, 47). Modulation of interferon signaling has not previously been reported for US28, but in the context of the latently infected monocyte, a general block in downstream interferon signaling may be important for maintaining the polarization of the monocyte (71, 72) or perhaps to avoid the antiviral activities of ISGs. We believe these questions merit further interrogation.

We chose to focus on the two PYHIN proteins and the set of HLA-DR components which are downregulated by US28. We confirmed downregulation of IFI16, MNDA, and HLA-DR in THP-1 cells which overexpress US28 and recapitulated these effects in experimental latency in primary CD14^+^ monocytes. HLA-DR was previously reported to be downregulated during experimental latency in granulocyte-macrophage progenitor cells, which prevents CD4^+^ T cell recognition and activation (41, 73, 74). While this downregulation of MHC class II involved the expression of the latency-associated gene UL111A (41), our data argue that viral US28 could also contribute to this phenotype. Little is known about the function of MNDA, a myeloid-specific PYHIN protein implicated in neutrophil cell death (75) and monocyte transcriptional networks (76). Ongoing work in our laboratory aims to identify whether US28-mediated downregulation of MNDA during latent infection could benefit latent carriage and/or reactivation.

Our results clearly characterized a rapid downregulation of IFI16 during the establishment of latency in monocytes, which occurred within the first 24 h of infection and was also maintained during long-term latency in monocytes and CD34^+^ HPCs. The early downregulation of IFI16 was clearly US28 dependent, as ΔUS28 virus failed to display immediate IFI16 downregulation. However, we did observe a partial downregulation of IFI16 in ΔUS28-infected monocytes at later time points of infection. We think it likely that this involves an unidentified lytic-phase viral gene product, which may be required for overcoming the known IFI16-mediated restriction of HCMV lytic infection (30, 34, 36, 37) and occurs as a result of ΔUS28 virus initiating a lytic infection in undifferentiated monocytes.

Our observation that the US28-dependent downregulation of IFI16 occurred rapidly (within 24 h of infection) may, in part, be attributable to incoming US28 which is functional (25). IFI16 protein has a short half-life of approximately 150 min in fibroblasts (77), and therefore, incoming US28 protein may rapidly target IFI16 transcription in latently infected monocytes, as it does in US28-expressing THP-1 cells, resulting in loss of IFI16 within 24 h of infection; this is then maintained by subsequent latency-associated *de novo* US28 expression.

We found that preventing IFI16 expression has a clear benefit to the establishment of HCMV latency. This contrasts with previous analyses of latency in other viral systems, where IFI16 expression is necessary to repress lytic viral transcription (39, 40). In our study, IFI16 overexpression activated MIEP activity in the absence of additional viral proteins, and furthermore, IFI16 overexpression increased IE-positive nuclei in latently infected THP-1 cells. IFI16 activates the MIEP during lytic infection (32, 34), though in these cases an additional viral gene product, UL83, is thought to be required. Our results suggest that UL83 is not required for IFI16-mediated activation of the MIEP in undifferentiated myeloid cells and suggest that IFI16 activates NF-κB to achieve this, as use of either an NF-κB pathway inhibitor or deletion of NF-κB binding sites from the MIEP prevented IFI16-mediated IE expression. We believe this provides one mechanism by which US28 blocks NF-κB activity early during latency, a phenomenon we previously showed to be important for the establishment of latency in myeloid cells (21).

Taken together, our results suggest that one of the early events in the establishment of latency in CD14^+^ monocytes is the US28-mediated targeting of interferon-responsive genes, including the downregulation of IFI16, which serves to support the repression of the MIEP.

## MATERIALS AND METHODS

### Cells.

All cells were maintained at 37°C in a 5% CO_2_ atmosphere. THP-1 cells (European Collection of Authenticated Cell Cultures [ECACC] 88081201) were cultured in RPMI 1640 medium (Sigma) supplemented with 10% heat-inactivated fetal bovine serum (FBS) (PAN Biotech), 100 U/ml penicillin and 100 μg/ml streptomycin (Sigma), and 0.05 mM 2-mercaptoethanol (Gibco). Kasumi-3 cells (ATCC CRL-2725) were cultured in RPMI 1640 medium (Sigma) supplemented with 20% heat-inactivated FBS (PAN Biotech) and 100 U/ml penicillin and 100 μg/ml streptomycin (Sigma). During infections, THP-1 and Kasumi-3 cells were cultured in a low-serum (1%) version of this medium for a minimum of 24 h prior to inoculation and maintained in this low-serum medium throughout the infection. MIEP-eGFP reporter THP-1 cells (63) were a gift from M. Van Loock (Johnson & Johnson). RPE-1 cells (ATCC CRL-4000) and human foreskin fibroblasts (Hff1; ATCC SCRC-1041) were maintained in Dulbecco modified Eagle medium DMEM (Sigma) supplemented with 10% heat-inactivated FBS and 100 U/ml penicillin and 100 μg/ml streptomycin. 293T cells (ECACC 12022001) were maintained in DMEM (Sigma) supplemented with 10% heat-inactivated FBS but without penicillin or streptomycin. Phorbol 12-myristate 13-acetate (PMA) (Sigma) was used to induce myeloid cell differentiation at a concentration of 20 ng/ml.

Primary CD14^+^ monocytes were isolated from apheresis cones (National Health Services [NHS] Blood & Transplant Service) or from peripheral blood samples taken from healthy volunteers as previously described (78). Briefly, CD14^+^ monocytes were isolated from total peripheral blood mononuclear cells (PBMC) by magnetically activated cell sorting (MACS) using CD14^+^ microbeads (Miltenyi Biotech). The monocytes were plated on tissue culture dishes (Corning) or slides (Ibidi), or kept in suspension in X-Vivo 15 medium (Lonza) supplemented with 2 mM l-glutamine. Mature dendritic cells were generated by treating CD14^+^ monocytes with granulocyte-macrophage colony-stimulating factor (GM-CSF) (Miltenyi) (1,000 U/ml) and interleukin-4 (IL-4) (Miltenyi) (1,000 U/ml) for 5 days before the addition of lipopolysaccharide (LPS) (Invivogen) (50 ng/ml) for 2 further days.

Primary human CD34^+^ hematopoietic progenitor cells, isolated from adult bone marrow, were purchased from STEMCELL Technologies and cultured in X-Vivo 15 medium (Lonza).

### Ethics statement.

All human samples were obtained under ethical approval and after approval of protocols from the Cambridgeshire 2 Research Ethics Committee (REC reference 97/092), and all protocols were conducted in accordance with the Declaration of Helsinki. Informed written consent was obtained from all of the volunteers included in this study prior to providing blood samples, and all experiments were conducted in accordance with the approved guidelines.

### Generation of lentiviruses and retroviruses.

The lentiviral vectors encoding US28 from the VHL/E strain of human cytomegalovirus (HCMV) have been described previously (21); US28 is expressed in these vectors via the spleen focus-forming virus (SFFV) promoter. The lentiviral vectors pHRSIN UbEm and pHRsin SV40blast were a kind gift from D. van den Boomen, University of Cambridge, and were based upon a previously published lentiviral system (79, 80). Briefly, expression of the gene of interest is also driven by the SFFV promoter, and the selectable markers Emerald and blasticidin resistance are driven by the ubiquitin promoter (UbEm) and the SV40 promoter (SV40blast), respectively. The sequence encoding US28 from the VHL/E strain of HCMV was cloned into pHRSIN UbEm using the EcoRI and SpeI sites using the following primers: US28 FW (FW stands for forward), 5′ GCAcgaattccatatgacgccgacgacgac, and RV (RV stands for reverse), 5′-CTGCACTAGTTTACGGTATAATTTGTGAGAC. The sequence encoding IFI16 was cloned into pHRsin SV40blast using the BamHI and NotI sites using the following primers: IFI16 FW, 5′-GATTGCGGCCGCATGGGAAAAAAATACAAGAACATTGTTC and RV, 5′-GATCGGATCCTTAGAAGAAAAAGTCTGGTGAAGTTTC.

The sequence encoding US28-3XFLAG was cloned from TB40/E*mCherry*-US28-3XFLAG into the retroviral plasmid pBABE eGFP (a gift from Debu Chakravarti [Addgene plasmid no. 36999]) as described previously (25). The Q5 site-directed mutagenesis kit (New England Biotech) was used to generate the US28-R129A mutant of this vector, which was verified by Sanger sequencing. Expression of US28 in these vectors is driven by the long terminal repeat and partial gag.

Generation of vesicular stomatitis virus G protein (VSV-G) pseudotyped lentiviral particles was conducted generally in line with the Broad Institute Protocols. Five hundred thousand 293T cells were transfected with 1,250 ng of lentiviral expression vector, 625 ng of lentiviral packaging vector psPAX, and 625 ng of envelope vector pMD.2G (both gifts from S. Karniely, Weizmann Institute, Israel) using transfection reagent FuGene6 (Promega) according to the manufacturer’s instructions. For generation of VSV-G pseudotyped retrovirus particles, 1,250 ng of the murine leukemia virus retroviral packaging vector KB4 (81) (a gift from H. Groom, University of Cambridge) was transfected along with 625 ng pMD.2G and 1250 ng retroviral expression vector.

### Lentiviral and retroviral transduction.

Supernatants from transfected 293T cells were harvested at 36 and 60 h posttransfection, filtered through a 0.45-μm syringe filter, and used to transduce THP-1 cells in the presence of 2 μg/ml Polybrene. When necessary, lentiviral titers were determined by in-house p24 enzyme-linked immunosorbent assay (ELISA). For transduction with puromycin resistance vectors, puromycin (2 μg/ml, Sigma) was added to media and refreshed every 2 to 5 days until all control nontransduced THP-1 cells were dead. Similarly, where blasticidin resistance vectors were used, blasticidin (1 μg/ml; Invivogen) was added to media. Emerald-positive cells were sorted using a BD FACSAriaIII instrument.

### Human cytomegaloviruses.

Infection of monocytes and THP-1 cells was conducted at a multiplicity of infection (MOI) of 3 as determined by titration on RPE-1 cells. TB40/E BAC4 strains were propagated in RPE-1 cells by seeding 50% confluent T175 flasks with virus at an MOI of 0.1. Spread of virus was monitored for 2 to 6 weeks following inoculation by fluorescence microscopy, and infected monolayers were subcultured twice during this period. When cells were 95 to 100% infected, supernatants were harvested on three occasions spaced over 7 days and stored at –80°C. In the final harvest, the monolayer was scraped and also stored at –80°C. After thawing the virus-containing media, cell debris was pelleted by centrifugation at 1,500 × *g* for 20 min at room temperature. Then, the clarified supernatant was concentrated by high-speed centrifugation at 14,500 × *g* for 2 h at 18°C. Virus-containing pellets were then resuspended in X-Vivo 15 medium in aliquots at –80°C.

TB40/E*mCherry*-US28-3XFLAG and TB40/E*mCherry*-US28Δ have been described previously (19). TB40/E*gfp* (82) and TB40/E BAC4 SV40 mCherry IE2-2A-GFP (83) were kind gifts from E. A. Murphy, SUNY Upstate Medical University. TB40/E BAC4 IE2-eYFP has been described previously (60, 84). TB40/E BAC4 GATA2mCherry has been described previously (50). TB40/E with deleted NF-κB sites in the MIEP at positions −94, −157, −262, and −413 and the revertant virus were a kind gift from Jeffery Meier and Ming Li (University of Iowa, United States) and have been described previously (65).

UV inactivation of virus was performed by placing a 100-μl aliquot of virus in one well of a 24-well plate and placing this within 10 cm of a UV germicidal (254-nm) lamp for 15 min, which routinely results in no detectable IE gene expression upon infection of Hff1 cells.

### Immunofluorescence staining and image analysis.

Cells were fixed with 2% paraformaldehyde for 15 min and permeabilized with 0.1% Triton X-100 for 10 min at room temperature. Blocking and antibody incubations were performed in phosphate-buffered saline (PBS) with 1% bovine serum albumin and 5% normal goat serum. The following antibodies were used: anti-IFI16 (Santa Cruz sc-8023, 1:100), anti-FLAG (Sigma F1804, 1:1000), anti-MNDA (Cell Signaling Technology 3329, 1:100), anti-IE (Argene 11-003, 1:1,000 or directly conjugated to fluorescein isothiocyanate [FITC], 1:100), anti-GFP (directly conjugated to FITC, Abcam ab6662, 1:200), anti-mCherry (Abcam ab167453, 1:500), anti-HLA DR (conjugated to Brilliant Violet 421, Biolegend clone L423 or Abcam ab92511, 1:100). Cells were imaged with a wide-field Nikon TE200 microscope, and images were processed using ImageJ. For contingency analyses of IFI16 expression during experimental latency, cells were assigned as “IFI16 positive/negative” and “infected/uninfected” and then counted. These results were then analyzed using Fisher’s exact statistical test for significance. For analysis of signal intensity, nuclear stained images were used to create a mask from which intensity values of the corresponding IFI16/MNDA-stained image were derived using the Analyze Particles feature of ImageJ. Cells were assigned as infected or uninfected based on signal from the GFP/mCherry stain. The average signal intensity of uninfected cells was used to normalize the signal intensity in order to compare different fields of view.

### Inhibitors.

The c-*fos* inhibitor T5524 was purchased from Cayman Chemical, solubilized in dimethyl sulfoxide (DMSO) and used at 10 μM. The Janus kinase inhibitor Ruxolitinib was purchased from Cell Guidance Systems, solubilized in DMSO and used at 5 μM. The IκB kinase alpha (IKKα) inhibitor/NF-κB pathway inhibitor BAY11-7082 was purchased from Santa Cruz, solubilized in DMSO, and used at a concentration of 5 μM.

### Western blotting.

Except for US28 blots, cells were lysed directly in Laemmli buffer and separated by sodium dodecyl sulfate-polyacrylamide gel electrophoresis (SDS-PAGE). Following transfer to nitrocellulose, the membrane was blocked in 5% milk in Tris-buffered saline (TBS) with 0.1% Tween 20. The following antibodies were used: anti-IFI16 (Santa Cruz sc-8023, 1:500), anti-MNDA (Cell Signaling Technology 3329, 1:250), anti-STAT1 (Cell Signaling Technology 14994, 1:1,000), anti-phosphoSTAT1 Tyr701 (Cell Signaling Technology 9167, 1:1,000), and anti-beta actin (Abcam ab6276, 1:5,000). For US28 blots, cells were pelleted, washed once in ice-cold PBS, and then lysed in native lysis buffer (25 mM Tris HCl [pH 7.4], 150 mM NaCl, 1 mM EDTA, 1% NP-40, 5% glycerol, plus protease inhibitors) for 30 min, vortexing every 10 min. After the addition of nonreducing Laemmli buffer, samples were heated at 42°C for 10 min and then separated by SDS-PAGE. Polyvinylidene difluoride membranes were used for transfer, and blocked membranes were incubated with the rabbit anti-US28 antibody (85) (a gift from M. Smit, Vrije University) at 1:1,000 dilution. To quantify Western blots, the Analyze Gels feature of Image J was used to plot the band intensities. The signals for genes of interest were then normalized to those of US28-R129A cells and then normalized to either the relative amounts of actin or STAT1 as described in the figure legends.

### Flow cytometry.

Transduced THP-1 cells and MIEP reporter THP-1 cells were analyzed on a BD Accuri instrument. Live cells were gated using forward and side scatter. Paraformaldehyde-fixed cells were stained using anti-HLA-DR allophycocyanin (APC) conjugate (Biolegend, clone L243, 1:50). Latently infected CD14^+^ monocytes were fixed with 1% paraformaldehyde and stained using anti-HLA-DR Pacific blue conjugate (Biolegend, clone L243, 1:50) and anti-HLA-A,B,C, phycoerythrin (PE)-Cy7 conjugate (Biolegend, clone W6/32, 1:50), before analysis on the Nxt Attune instrument (Thermo Fisher Scientific).

### RNA extraction, reverse transcription, and quantitative PCR.

RNA was extracted and purified using Direct-Zol RNA MiniPrep kit (Zymo Research) according to the manufacturer’s instructions. A total of 5 ng of purified RNA was used in reverse transcription-quantitative PCR (RT-qPCR), performed using QuantiTect SYBR Green RT-PCR kit reagents (Qiagen) on a StepOne Real-Time PCR instrument (Applied Biosystems). TATA box binding protein (TBP) was used as a reference gene, and fold changes were analyzed by the 2^−ΔΔCt^ method. Reverse transcription was performed using the Qiagen QuantiTect Reverse Transcription kit, and then cDNA was used in qPCR analysis using New England Biotech LUNA SYBR green qPCR reagents using TBP or glyceraldehyde-3-phosphate dehydrogenase (GAPDH) as a reference gene. The following primers were used: US28 FW, AATCGTTGCGGTGTCTCAGT; US28 RV, TGGTACGGCAGCCAAAAGAT; MNDA FW, GGAAGAAGCATCCATTAAGG; MNDA RV, GTTTGTCTAGACAGGCAAC; IFI16 FW, CTGCACCCTCCACAAG; IFI16 RV, CCATGGCTGTGGACATG; TBP FW, CGGCTGTTTAACTTCGCTTC; TBP RV, CACACGCCAAGAAACAGTGA; HLA-DRA FW, TGTAAGGCACATGGAGGTGA; HLA-DRA RV, ATAGGGCTGGAAAATGCTGA; IE72 FW, GTCCTGACAGAACTCGTCAAA; IE72 RV, TAAAGGCGCCAGTGAATTTTTCTTC; GAPDH FW, TGCACCACCAACTGCTTAGC; and GAPDH RV, GGCATGGACTGTGGTCATGAG.

### Sequence alignment.

US28 sequences were exported from GenBank (TB40/E BAC4 EF999921.1, AD169 FJ527563.1, Merlin NC_006273.2, and VHL/E MK425187.1) or from sequenced plasmids and aligned using Clustal Omega (86) (https://www.ebi.ac.uk/Tools/msa/clustalo/) and the output format MView (87).

### Cell lysis, digestion, and cleanup for proteomic analysis.

Cells were harvested by centrifugation and washed three times in cold phosphate-buffered saline before finally pelleting into a low-adhesion microcentrifuge tube. Cell pellets were lysed in 2% 50 mM triethylammonium bicarbonate (TEAB) (pH 8.5). Samples were quantified by bicinchoninic acid (BCA) assay, and 50 μg of each sample was taken and adjusted to the same volume with lysis buffer. Reduction and alkylation were achieved by the addition of 10 mM Tris (2-carboxyethyl)phosphine (TCEP) and 20 mM iodoacetamide for 20 min at room temperature in the dark followed by quenching with 10 mM dithiothreitol (DTT). Samples were further purified and digested using a modified filtered aided sample prep (FASP) protocol. Briefly, samples were brought to 500-μl volume with 8 M urea−TEAB and loaded onto a 30-kDa molecular-weight-cutoff (MWCO) ultrafiltration device. The samples were then washed three times with 8 M urea−TEAB and then washed three times with 0.5% sodium deoxycholate (SDC)−50 mM TEAB. Samples were finally resuspended in ∼50 μl of SDC−TEAB containing 1 μg trypsin and incubated overnight at 37°C. After digestion, samples were recovered from the filter device by centrifugation, acidified to precipitate SDC and cleaned up by two-phase partitioning into 2× volumes of ethyl acetate (repeated twice) before drying in a vacuum centrifuge.

### TMT labeling.

Samples were resuspended in 20 μl of 100 mM TEAB, and to each tube 0.2 μg of a unique tandem mass tag (TMT) label for each sample was added in 8.5 μl acetonitrile (ACN) and incubated for 1 h at room temperature. TMT reactions were quenched by the addition of 3 μl of 200 mM ammonium formate, pooled, and dried in a vacuum centrifuge. The sample was then resuspended in 800 μl of 0.1% trifluoroacetic acid (TFA) and acidified to ∼pH 2 with formic acid (FA) before performing a C18 solid-phase extraction (C18-SPE) using a Sep-Pak cartridge (Waters) attached to a vacuum manifold. C18 eluate was dried in a vacuum centrifuge and resuspended in 40 μl od 200 mM ammonium formate, pH 10.

### High-pH reverse-phase fractionation.

The samples were injected onto an Ultimate 3000 RSLC UHPLC system (Thermo Fisher Scientific) equipped with a 2.1 mm inner diameter (i.d.) × 25 cm, 1.7-μm particle Kinetix Evo C_18_ column (Phenomenex). The mobile phase consisted of solvent A (3% ACN), solvent B (ACN), and solvent C (200 mM ammonium formate [pH 10]). Isocratic conditions were 90% solvent A−10% solvent C, and solvent C was maintained at 10% throughout the gradient elution. Separations were conducted at 45°C. After loading at 200 μl/min for 5 min and ramping the flow rate to 400 μl/min over 5 min, the gradient elution proceeded as follows: 0 to 19% solvent B over 10 min (curve 3), 19 to 34% solvent B over 14.25 min (curve 5), 34 to 50% solvent B over 8.75 min (curve 5), followed by a 10-min wash with 90% solvent B. UV absorbance was monitored at 280 nm, and 15-second fractions were collected into 96-well microplates using the integrated fraction collector. Peptide containing fractions were then orthogonally recombined into 24 fractions, dried in a vacuum centrifuge, and resuspended in 10 μl of 5% DMSO−0.5% TFA for analysis.

### Liquid chromatography (LC)-mass spectrometry (MS) analysis.

All samples were injected onto an Ultimate 3000 RSLC nano UHPLC equipped with a 300-μm i.d. × 5-mm Acclaim PepMap μ-Precolumn (Thermo Fisher Scientific) and a 75-μm i.d. × 50-cm 2.1-μm particle Acclaim PepMap RSLC analytical column. The loading solvent was 0.1% TFA, analytical solvent A was 0.1% FA, and analytical solvent B was ACN plus 0.1% FA. All separations are conducted at 55°C. Samples were loaded at 10 μl/min for 5 min in loading solvent before beginning the analytical gradient. For high-pH reverse-phase (RP) fractions, a gradient of 3 to 5.6% analytical solvent B over 4 min, 5.6 to 32% analytical solvent B over 162 min, followed by a 5-min wash with 80% analytical solvent B, a 5-min wash with 90% analytical solvent B, and equilibration at 3% analytical solvent B for 5 min. During the gradient, the Orbitrap Fusion mass spectrometer (Thermo Fisher Scientific) was set to acquire spectra according to the settings given in Table S1 in the supplemental material (in “MS Settings”).

10.1128/mBio.02574-19.6 TABLE S1 Schematic showing mass spectrometry settings for the experiments presented in Fig. 1 and Data Set S1. Download Table S1, PDF file, 0.2 MB. Copyright © 2019 Elder et al. 2019 Elder et al. This content is distributed under the terms of the Creative Commons Attribution 4.0 International license.

### Data processing.

All raw files were searched by Mascot within Proteome Discoverer 2.1 (Thermo Fisher Scientific) against the Swissprot human database and a database of common contaminants.

The search parameters were as follows: enzyme, trypsin; MS1 tolerance (tol), 10 ppm; MS2 tol, 0.6 Da; fixed modifications, carbamidomethyl cysteine, TMT peptide N termini and lysine; variable modification, oxidized methionine; MS3 reporter ion tol, 20 ppm, most confident centroid. Mascot Percolator was used to calculate peptide spectrum match false-discovery rate (PSM FDR).

Search results were further processed and filtered as follows. Peptides below a percolator FDR of 0.01% and proteins below the 0.01% protein FDR (calculated from a built-in decoy database search) were rejected. Protein groups were then generated using the strict parsimony principle. Peptides both unique and razor with a coisolation threshold of 50 and an average signal-to-noise (s/n) threshold of 10 were used for quantification, and a normalization of these values to the total peptide amount in each channel was applied. Instances where a protein was identified but not quantified in all channels were rejected from further analysis. “Scaled” abundances of proteins provided by Proteome Discoverer were used to derive ratios of abundance. *q* values of significance between groups were calculated by Benjamini-Hochberg correction of *P* values generated using the moderated T-test LIMMA within the R environment.

## References

[1] Hahn G, Jores R, Mocarski ES 1998 Cytomegalovirus remains latent in a common precursor of dendritic and myeloid cells. Proc Natl Acad Sci U S A 95:3937–3942. doi:10.1073/pnas.95.7.3937. 9520471 PMC19941

[2] Kondo K, Xu J, Mocarski ES 1996 Human cytomegalovirus latent gene expression in granulocyte-macrophage progenitors in culture and in seropositive individuals. Proc Natl Acad Sci U S A 93:11137–11142. doi:10.1073/pnas.93.20.11137. 8855322 PMC38297

[3] Taylor-Wiedeman J, Sissons JGP, Borysiewicz LK, Sinclair JH 1991 Monocytes are a major site of persistence of human cytomegalovirus in peripheral blood mononuclear cells. J Gen Virol 72:2059–2064. doi:10.1099/0022-1317-72-9-2059. 1654370

[4] Mendelson M, Monard S, Sissons P, Sinclair J 1996 Detection of endogenous human cytomegalovirus in CD34+ bone marrow progenitors. J Gen Virol 77:3099–3102. doi:10.1099/0022-1317-77-12-3099. 9000102

[5] Reeves MB, Sinclair JH 2013 Circulating dendritic cells isolated from healthy seropositive donors are sites of human cytomegalovirus reactivation in vivo. J Virol 87:10660–10667. doi:10.1128/JVI.01539-13. 23885077 PMC3807413

[6] Poole E, Juss JK, Krishna B, Herre J, Chilvers ER, Sinclair J 2015 Alveolar macrophages isolated directly from human cytomegalovirus (HCMV)-seropositive individuals are sites of HCMV reactivation in vivo. J Infect Dis 211:1936–1942. doi:10.1093/infdis/jiu837. 25552371 PMC4442624

[7] Reeves MB, Breidenstein A, Compton T 2012 Human cytomegalovirus activation of ERK and myeloid cell leukemia-1 protein correlates with survival of latently infected cells. Proc Natl Acad Sci U S A 109:588–593. doi:10.1073/pnas.1114966108. 22203987 PMC3258610

[8] Reeves MB, Lehner PJ, Sissons JGP, Sinclair JH 2005 An in vitro model for the regulation of human cytomegalovirus latency and reactivation in dendritic cells by chromatin remodelling. J Gen Virol 86:2949–2954. doi:10.1099/vir.0.81161-0. 16227215

[9] Jackson SE, Mason GM, Wills MR 2011 Human cytomegalovirus immunity and immune evasion. Virus Res 157:151–160. doi:10.1016/j.virusres.2010.10.031. 21056604

[10] Chan ST, Logan AC 2017 The clinical impact of cytomegalovirus infection following allogeneic hematopoietic cell transplantation: why the quest for meaningful prophylaxis still matters. Blood Rev 31:173–183. doi:10.1016/j.blre.2017.01.002. 28173959

[11] Carbone J 2016 The immunology of posttransplant CMV infection. Transplantation 100:S11–S18. doi:10.1097/TP.0000000000001095. 26900990 PMC4764014

[12] Reeves MB, MacAry PA, Lehner PJ, Sissons JGP, Sinclair JH 2005 Latency, chromatin remodeling, and reactivation of human cytomegalovirus in the dendritic cells of healthy carriers. Proc Natl Acad Sci U S A 102:4140–4145. doi:10.1073/pnas.0408994102. 15738399 PMC554799

[13] Goodrum FD, Jordan CT, High K, Shenk T 2002 Human cytomegalovirus gene expression during infection of primary hematopoietic progenitor cells: a model for latency. Proc Natl Acad Sci U S A 99:16255–16260. doi:10.1073/pnas.252630899. 12456880 PMC138598

[14] Söderberg-Nauclér C, Fish KN, Nelson JA 1997 Reactivation of latent human cytomegalovirus by allogeneic stimulation of blood cells from healthy donors. Cell 91:119–126. doi:10.1016/s0092-8674(01)80014-3. 9335340

[15] Stinski MF, Isomura H 2008 Role of the cytomegalovirus major immediate early enhancer in acute infection and reactivation from latency. Med Microbiol Immunol 197:223–231. doi:10.1007/s00430-007-0069-7. 18097687

[16] Nelson JA, Groudine M 1986 Transcriptional regulation of the human cytomegalovirus major immediate-early gene is associated with induction of DNase I-hypersensitive sites. Mol Cell Biol 6:452–461. doi:10.1128/MCB.6.2.452. 3023848 PMC367533

[17] Boshart M, Weber F, Jahn G, Dorsch-Häsler K, Fleckenstein B, Schaffner W 1985 A very strong enhancer is located upstream of an immediate early gene of human cytomegalovirus. Cell 41:521–530. doi:10.1016/S0092-8674(85)80025-8. 2985280

[18] Elder E, Sinclair J 2019 HCMV latency: what regulates the regulators? Med Microbiol Immunol 208:431. doi:10.1007/s00430-019-00581-1. 30761409 PMC6647427

[19] Humby MS, O’Connor CM 2015 Human cytomegalovirus US28 is important for latent infection of hematopoietic progenitor cells. J Virol 90:2959–2970. doi:10.1128/JVI.02507-15. 26719258 PMC4810657

[20] Beisser PS, Laurent L, Virelizier J-L, Michelson S 2001 Human cytomegalovirus chemokine receptor gene US28 is transcribed in latently infected THP-1 monocytes. J Virol 75:5949–5957. doi:10.1128/JVI.75.13.5949-5957.2001. 11390596 PMC114310

[21] Krishna BA, Poole EL, Jackson SE, Smit MJ, Wills MR, Sinclair JH 2017 Latency-associated expression of human cytomegalovirus US28 attenuates cell signaling pathways to maintain latent infection. mBio 8:e01754-17. doi:10.1128/mBio.01754-17. 29208743 PMC5717388

[22] Hargett D, Shenk TE 2010 Experimental human cytomegalovirus latency in CD14+ monocytes. Proc Natl Acad Sci U S A 107:20039–20044. doi:10.1073/pnas.1014509107. 21041645 PMC2993366

[23] Zhu D, Pan C, Sheng J, Liang H, Bian Z, Liu Y, Trang P, Wu J, Liu F, Zhang C-Y, Zen K 2018 Human cytomegalovirus reprogrammes haematopoietic progenitor cells into immunosuppressive monocytes to achieve latency. Nat Microbiol 3:503–513. doi:10.1038/s41564-018-0131-9. 29588542 PMC6537872

[24] Krishna BA, Spiess K, Poole EL, Lau B, Voigt S, Kledal TN, Rosenkilde MM, Sinclair JH 2017 Targeting the latent cytomegalovirus reservoir with an antiviral fusion toxin protein. Nat Commun 8:14321. doi:10.1038/ncomms14321. 28148951 PMC5296658

[25] Krishna BA, Humby MS, Miller WE, O’Connor CM 2019 Human cytomegalovirus G protein-coupled receptor US28 promotes latency by attenuating c-fos. Proc Natl Acad Sci U S A 116:1755–1764. doi:10.1073/pnas.1816933116. 30647114 PMC6358704

[26] Krishna BA, Miller WE, O’Connor CM 2018 US28: HCMV’s Swiss Army knife. Viruses 10:E445. doi:10.3390/v10080445. 30127279 PMC6116241

[27] Orzalli MH, DeLuca NA, Knipe DM 2012 Nuclear IFI16 induction of IRF-3 signaling during herpesviral infection and degradation of IFI16 by the viral ICP0 protein. Proc Natl Acad Sci U S A 109:E3008-17. doi:10.1073/pnas.1211302109. 23027953 PMC3497734

[28] Dutta D, Dutta S, Veettil MV, Roy A, Ansari MA, Iqbal J, Chikoti L, Kumar B, Johnson KE, Chandran B 2015 BRCA1 regulates IFI16 mediated nuclear innate sensing of herpes viral DNA and subsequent induction of the innate inflammasome and interferon-β responses. PLoS Pathog 11:e1005030. doi:10.1371/journal.ppat.1005030. 26121674 PMC4487893

[29] Kerur N, Veettil MV, Sharma-Walia N, Bottero V, Sadagopan S, Otageri P, Chandran B 2011 IFI16 acts as a nuclear pathogen sensor to induce the inflammasome in response to Kaposi sarcoma-associated herpesvirus infection. Cell Host Microbe 9:363–375. doi:10.1016/j.chom.2011.04.008. 21575908 PMC3113467

[30] Li T, Chen J, Cristea IM 2013 Human cytomegalovirus tegument protein pUL83 inhibits IFI16-mediated DNA sensing for immune evasion. Cell Host Microbe 14:591–599. doi:10.1016/j.chom.2013.10.007. 24237704 PMC3876934

[31] Alandijany T, Roberts APE, Conn KL, Loney C, McFarlane S, Orr A, Boutell C 2018 Distinct temporal roles for the promyelocytic leukaemia (PML) protein in the sequential regulation of intracellular host immunity to HSV-1 infection. PLoS Pathog 14:e1006769. doi:10.1371/journal.ppat.1006769. 29309427 PMC5757968

[32] Cristea IM, Moorman NJ, Terhune SS, Cuevas CD, O’Keefe ES, Rout MP, Chait BT, Shenk T 2010 Human cytomegalovirus pUL83 stimulates activity of the viral immediate-early promoter through its interaction with the cellular IFI16 protein. J Virol 84:7803–7814. doi:10.1128/JVI.00139-10. 20504932 PMC2897612

[33] Diner BA, Lum KK, Toettcher JE, Cristea IM 2016 Viral DNA sensors IFI16 and cyclic GMP-AMP synthase possess distinct functions in regulating viral gene expression, immune defenses, and apoptotic responses during herpesvirus infection. mBio 7:e01553-16. doi:10.1128/mBio.01553-16. 27935834 PMC5111403

[34] Gariano GR, Dell’Oste V, Bronzini M, Gatti D, Luganini A, De Andrea M, Gribaudo G, Gariglio M, Landolfo S 2012 The intracellular DNA sensor IFI16 gene acts as restriction factor for human cytomegalovirus replication. PLoS Pathog 8:e1002498. doi:10.1371/journal.ppat.1002498. 22291595 PMC3266931

[35] Diner BA, Lum KK, Javitt A, Cristea IM 2015 Interactions of the antiviral factor interferon gamma-inducible protein 16 (IFI16) mediate immune signaling and herpes simplex virus-1 immunosuppression. Mol Cell Proteomics 14:2341–2356. doi:10.1074/mcp.M114.047068. 25693804 PMC4563720

[36] Johnstone RW, Kerry JA, Trapani JA 1998 The human interferon-inducible protein, IFI 16, is a repressor of transcription. J Biol Chem 273:17172–17177. doi:10.1074/jbc.273.27.17172. 9642285

[37] Biolatti M, Dell’Oste V, Pautasso S, von Einem J, Marschall M, Plachter B, Gariglio M, De Andrea M, Landolfo S 2016 Regulatory interaction between the cellular restriction factor IFI16 and viral pp65 (pUL83) modulates viral gene expression and IFI16 protein stability. J Virol 90:8238–8250. doi:10.1128/JVI.00923-16. 27384655 PMC5008087

[38] Roy A, Dutta D, Iqbal J, Pisano G, Gjyshi O, Ansari MA, Kumar B, Chandran B 2016 Nuclear innate immune DNA sensor IFI16 is degraded during lytic reactivation of Kaposi’s sarcoma-associated herpesvirus (KSHV): role of IFI16 in maintenance of KSHV latency. J Virol 90:8822–8841. doi:10.1128/JVI.01003-16. 27466416 PMC5021400

[39] Pisano G, Roy A, Ahmed Ansari M, Kumar B, Chikoti L, Chandran B 2017 Interferon-γ-inducible protein 16 (IFI16) is required for the maintenance of Epstein-Barr virus latency. Virol J 14:221. doi:10.1186/s12985-017-0891-5. 29132393 PMC5683537

[40] Hotter D, Bosso M, Jønsson KL, Krapp C, Stürzel CM, Das A, Littwitz-Salomon E, Berkhout B, Russ A, Wittmann S, Gramberg T, Zheng Y, Martins LJ, Planelles V, Jakobsen MR, Hahn BH, Dittmer U, Sauter D, Kirchhoff F 2019 IFI16 targets the transcription factor Sp1 to suppress HIV-1 transcription and latency reactivation. Cell Host Microbe 25:858–872.e13. doi:10.1016/j.chom.2019.05.002. 31175045 PMC6681451

[41] Cheung AKL, Gottlieb DJ, Plachter B, Pepperl-Klindworth S, Avdic S, Cunningham AL, Abendroth A, Slobedman B 2009 The role of the human cytomegalovirus UL111A gene in down-regulating CD4+ T-cell recognition of latently infected cells: implications for virus elimination during latency. Blood 114:4128–4137. doi:10.1182/blood-2008-12-197111. 19706889

[42] Rusinova I, Forster S, Yu S, Kannan A, Masse M, Cumming H, Chapman R, Hertzog PJ 2013 Interferome v2.0: an updated database of annotated interferon-regulated genes. Nucleic Acids Res 41:D1040–D1046. doi:10.1093/nar/gks1215. 23203888 PMC3531205

[43] Rauch I, Müller M, Decker T 2013 The regulation of inflammation by interferons and their STATs. JAKSTAT 2:e23820. doi:10.4161/jkst.23820. 24058799 PMC3670275

[44] Ono SJ, Liou HC, Davidon R, Strominger JL, Glimcher LH 1991 Human X-box-binding protein 1 is required for the transcription of a subset of human class II major histocompatibility genes and forms a heterodimer with c-fos. Proc Natl Acad Sci U S A 88:4309–4312. doi:10.1073/pnas.88.10.4309. 1903538 PMC51648

[45] Ono SJ, Bazil V, Levi BZ, Ozato K, Strominger JL 1991 Transcription of a subset of human class II major histocompatibility complex genes is regulated by a nucleoprotein complex that contains c-fos or an antigenically related protein. Proc Natl Acad Sci U S A 88:4304–4308. doi:10.1073/pnas.88.10.4304. 1709740 PMC51647

[46] Lee YJ, Benveniste EN 1996 Stat1 alpha expression is involved in IFN-gamma induction of the class II transactivator and class II MHC genes. J Immunol 157:1559–1568. 8759739

[47] Clarke CJP, Apostolidis V, Hii LLP, Gough DJ, Trapani JA, Johnstone RW 2003 Critical role of the transcription factor AP-1 for the constitutive and interferon-induced expression of IFI 16. J Cell Biochem 89:80–93. doi:10.1002/jcb.10475. 12682910

[48] Dell’Oste V, Gatti D, Giorgio AG, Gariglio M, Landolfo S, De Andrea M 2015 The interferon-inducible DNA-sensor protein IFI16: a key player in the antiviral response. New Microbiol 38:5–20. 25742143

[49] Kristiansen M, Hughes R, Patel P, Jacques TS, Clark AR, Ham J 2010 Mkp1 is a c-Jun target gene that antagonizes JNK-dependent apoptosis in sympathetic neurons. J Neurosci 30:10820–10832. doi:10.1523/JNEUROSCI.2824-10.2010. 20702711 PMC3044878

[50] Elder E, Krishna B, Williamson J, Aslam Y, Farahi N, Wood A, Romashova V, Roche K, Murphy E, Chilvers E, Lehner PJ, Sinclair J, Poole E 2019 Monocytes latently infected with human cytomegalovirus evade neutrophil killing. iScience 12:13–26. doi:10.1016/j.isci.2019.01.007. 30677738 PMC6352302

[51] O’Connor CM, Murphy EA 2012 A myeloid progenitor cell line capable of supporting human cytomegalovirus latency and reactivation, resulting in infectious progeny. J Virol 86:9854–9865. doi:10.1128/JVI.01278-12. 22761372 PMC3446554

[52] Ansari MA, Singh VV, Dutta S, Veettil MV, Dutta D, Chikoti L, Lu J, Everly D, Chandran B 2013 Constitutive interferon-inducible protein 16-inflammasome activation during Epstein-Barr virus latency I, II, and III in B and epithelial cells. J Virol 87:8606–8623. doi:10.1128/JVI.00805-13. 23720728 PMC3719826

[53] Caposio P, Gugliesi F, Zannetti C, Sponza S, Mondini M, Medico E, Hiscott J, Young HA, Gribaudo G, Gariglio M, Landolfo S 2007 A novel role of the interferon-inducible protein IFI16 as inducer of proinflammatory molecules in endothelial cells. J Biol Chem 282:33515–33529. doi:10.1074/jbc.M701846200. 17699163

[54] Orzalli MH, Conwell SE, Berrios C, DeCaprio JA, Knipe DM 2013 Nuclear interferon-inducible protein 16 promotes silencing of herpesviral and transfected DNA. Proc Natl Acad Sci U S A 110:E4492–E4501. doi:10.1073/pnas.1316194110. 24198334 PMC3839728

[55] Thompson MR, Sharma S, Atianand M, Jensen SB, Carpenter S, Knipe DM, Fitzgerald KA, Kurt-Jones EA 2014 Interferon γ-inducible protein (IFI) 16 transcriptionally regulates type I interferons and other interferon-stimulated genes and controls the interferon response to both DNA and RNA viruses. J Biol Chem 289:23568–23581. doi:10.1074/jbc.M114.554147. 25002588 PMC4156042

[56] Johnson KE, Bottero V, Flaherty S, Dutta S, Singh VV, Chandran B 2014 IFI16 restricts HSV-1 replication by accumulating on the HSV-1 genome, repressing HSV-1 gene expression, and directly or indirectly modulating histone modifications. PLoS Pathog 10:e1004503. doi:10.1371/journal.ppat.1004503. 25375629 PMC4223080

[57] Song LL, Ponomareva L, Shen H, Duan X, Alimirah F, Choubey D 2010 Interferon-inducible IFI16, a negative regulator of cell growth, down-regulates expression of human telomerase reverse transcriptase (hTERT) gene. PLoS One 5:e8569. doi:10.1371/journal.pone.0008569. 20052289 PMC2797294

[58] Sponza S, De Andrea M, Mondini M, Gugliesi F, Gariglio M, Landolfo S 2009 Role of the interferon-inducible IFI16 gene in the induction of ICAM-1 by TNF-alpha. Cell Immunol 257:55–60. doi:10.1016/j.cellimm.2009.02.007. 19338980

[59] Sinclair J, Reeves M 2014 The intimate relationship between human cytomegalovirus and the dendritic cell lineage. Front Microbiol 5:389. doi:10.3389/fmicb.2014.00389. 25147545 PMC4124589

[60] Straschewski S, Warmer M, Frascaroli G, Hohenberg H, Mertens T, Winkler M 2010 Human cytomegaloviruses expressing yellow fluorescent fusion proteins--characterization and use in antiviral screening. PLoS One 5:e9174. doi:10.1371/journal.pone.0009174. 20161802 PMC2820100

[61] Lau B, Poole E, Van Damme E, Bunkens L, Sowash M, King H, Murphy E, Wills M, Van Loock M, Sinclair J 2016 Human cytomegalovirus miR-UL112-1 promotes the down-regulation of viral immediate early-gene expression during latency to prevent T-cell recognition of latently infected cells. J Gen Virol 97:2387–2398. doi:10.1099/jgv.0.000546. 27411311 PMC5756489

[62] Lee J-H, Kalejta RF 2019 Human cytomegalovirus enters the primary CD34+ hematopoietic progenitor cells where it establishes latency by macropinocytosis. J Virol 93:e00452-19. doi:10.1128/JVI.00452-19. 31118259 PMC6639267

[63] Van Damme E, Sauviller S, Lau B, Kesteleyn B, Griffiths P, Burroughs A, Emery V, Sinclair J, Van Loock M 2015 Glucocorticosteroids trigger reactivation of human cytomegalovirus from latently infected myeloid cells and increase the risk for HCMV infection in D+R+ liver transplant patients. J Gen Virol 96:131–143. doi:10.1099/vir.0.069872-0. 25312585 PMC4268819

[64] Dunphy G, Flannery SM, Almine JF, Connolly DJ, Paulus C, Jønsson KL, Jakobsen MR, Nevels MM, Bowie AG, Unterholzner L 2018 Non-canonical activation of the DNA sensing adaptor STING by ATM and IFI16 mediates NF-κB signaling after nuclear DNA damage. Mol Cell 71:745–760.e5. doi:10.1016/j.molcel.2018.07.034. 30193098 PMC6127031

[65] Kew V, Yuan J, Meier J, Reeves M 2014 Mitogen and stress activated kinases act co-operatively with CREB during the induction of human cytomegalovirus immediate-early gene expression from latency. PLoS Pathog 10:e1004195. doi:10.1371/journal.ppat.1004195. 24945302 PMC4055774

[66] Noriega VM, Gardner TJ, Redmann V, Bongers G, Lira SA, Tortorella D 2014 Human cytomegalovirus US28 facilitates cell-to-cell viral dissemination. Viruses 6:1202–1218. doi:10.3390/v6031202. 24625810 PMC3970146

[67] Vieira J, Schall TJ, Corey L, Geballe AP 1998 Functional analysis of the human cytomegalovirus US28 gene by insertion mutagenesis with the green fluorescent protein gene. J Virol 72:8158–8165. 9733857 10.1128/jvi.72.10.8158-8165.1998 PMC110158

[68] Zhang G, Zhang H, Liu Y, He Y, Wang W, Du Y, Yang C, Gao F 2014 CD44 clustering is involved in monocyte differentiation. Acta Biochim Biophys Sin (Shanghai) 46:540–547. doi:10.1093/abbs/gmu042. 24850301

[69] Schmid MA, Kingston D, Boddupalli S, Manz MG 2010 Instructive cytokine signals in dendritic cell lineage commitment. Immunol Rev 234:32–44. doi:10.1111/j.0105-2896.2009.00877.x. 20193010

[70] Watowich SS, Liu Y-J 2010 Mechanisms regulating dendritic cell specification and development. Immunol Rev 238:76–92. doi:10.1111/j.1600-065X.2010.00949.x. 20969586 PMC3039024

[71] Garrett S, Dietzmann-Maurer K, Song L, Sullivan KE 2008 Polarization of primary human monocytes by IFN-gamma induces chromatin changes and recruits RNA Pol II to the TNF-alpha promoter. J Immunol 180:5257–5266. doi:10.4049/jimmunol.180.8.5257. 18390706

[72] Chistiakov DA, Myasoedova VA, Revin VV, Orekhov AN, Bobryshev YV 2018 The impact of interferon-regulatory factors to macrophage differentiation and polarization into M1 and M2. Immunobiology 223:101–111. doi:10.1016/j.imbio.2017.10.005. 29032836

[73] Slobedman B, Mocarski ES, Arvin AM, Mellins ED, Abendroth A 2002 Latent cytomegalovirus down-regulates major histocompatibility complex class II expression on myeloid progenitors. Blood 100:2867–2873. doi:10.1182/blood.V100.8.2867. 12351397

[74] Jenkins C, Garcia W, Godwin MJ, Spencer JV, Stern JL, Abendroth A, Slobedman B 2008 Immunomodulatory properties of a viral homolog of human interleukin-10 expressed by human cytomegalovirus during the latent phase of infection. J Virol 82:3736–3750. doi:10.1128/JVI.02173-07. 18216121 PMC2268454

[75] Fotouhi-Ardakani N, Kebir DE, Pierre-Charles N, Wang L, Ahern SP, Filep JG, Milot E 2010 Role for myeloid nuclear differentiation antigen in the regulation of neutrophil apoptosis during sepsis. Am J Respir Crit Care Med 182:341–350. doi:10.1164/rccm.201001-0075OC. 20395555

[76] Suzuki T, Nakano-Ikegaya M, Yabukami-Okuda H, de Hoon M, Severin J, Saga-Hatano S, Shin JW, Kubosaki A, Simon C, Hasegawa Y, Hayashizaki Y, Suzuki H 2012 Reconstruction of monocyte transcriptional regulatory network accompanies monocytic functions in human fibroblasts. PLoS One 7:e33474. doi:10.1371/journal.pone.0033474. 22428058 PMC3302774

[77] Orzalli MH, Broekema NM, Knipe DM 2016 Relative contributions of herpes simplex virus 1 ICP0 and vhs to loss of cellular IFI16 vary in different human cell types. J Virol 90:8351–8359. doi:10.1128/JVI.00939-16. 27412599 PMC5008076

[78] Poole E, Reeves M, Sinclair JH 2014 The use of primary human cells (fibroblasts, monocytes, and others) to assess human cytomegalovirus function. Methods Mol Biol 1119:81–98. doi:10.1007/978-1-62703-788-4_6. 24639219

[79] Timms RT, Duncan LM, Tchasovnikarova IA, Antrobus R, Smith DL, Dougan G, Weekes MP, Lehner PJ 2013 Haploid genetic screens identify an essential role for PLP2 in the downregulation of novel plasma membrane targets by viral E3 ubiquitin ligases. PLoS Pathog 9:e1003772. doi:10.1371/journal.ppat.1003772. 24278019 PMC3836740

[80] van den Boomen DJH, Timms RT, Grice GL, Stagg HR, Skødt K, Dougan G, Nathan JA, Lehner PJ 2014 TMEM129 is a Derlin-1 associated ERAD E3 ligase essential for virus-induced degradation of MHC-I. Proc Natl Acad Sci U S A 111:11425–11430. doi:10.1073/pnas.1409099111. 25030448 PMC4128144

[81] Groom HCT, Boucherit VC, Makinson K, Randal E, Baptista S, Hagan S, Gow JW, Mattes FM, Breuer J, Kerr JR, Stoye JP, Bishop KN 2010 Absence of xenotropic murine leukaemia virus-related virus in UK patients with chronic fatigue syndrome. Retrovirology 7:10. doi:10.1186/1742-4690-7-10. 20156349 PMC2839973

[82] Spector DJ, Yetming K 2010 UL84-independent replication of human cytomegalovirus strain TB40/E. Virology 407:171–177. doi:10.1016/j.virol.2010.08.029. 20855098

[83] Poole E, Huang CJZ, Forbester J, Shnayder M, Nachshon A, Kweider B, Basaj A, Smith D, Jackson SE, Liu B, Shih J, Kiskin FN, Roche K, Murphy E, Wills MR, Morrell NW, Dougan G, Stern-Ginossar N, Rana AA, Sinclair J 2019 An iPSC-derived myeloid lineage model of herpes virus latency and reactivation. Front Microbiol 10:2233. doi:10.3389/FMICB.2019.02233. 31649625 PMC6795026

[84] Weekes MP, Tan SYL, Poole E, Talbot S, Antrobus R, Smith DL, Montag C, Gygi SP, Sinclair JH, Lehner PJ 2013 Latency-associated degradation of the MRP1 drug transporter during latent human cytomegalovirus infection. Science 340:199–202. doi:10.1126/science.1235047. 23580527 PMC3683642

[85] Slinger E, Maussang D, Schreiber A, Siderius M, Rahbar A, Fraile-Ramos A, Lira SA, Söderberg-Nauclér C, Smit MJ 2010 HCMV-encoded chemokine receptor US28 mediates proliferative signaling through the IL-6-STAT3 axis. Sci Signal 3:ra58. doi:10.1126/scisignal.2001180. 20682912

[86] Madeira F, Park YM, Lee J, Buso N, Gur T, Madhusoodanan N, Basutkar P, Tivey ARN, Potter SC, Finn RD, Lopez R 2019 The EMBL-EBI search and sequence analysis tools APIs in 2019. Nucleic Acids Res 47:W636–W641. doi:10.1093/nar/gkz268. 30976793 PMC6602479

[87] Brown NP, Leroy C, Sander C 1998 MView: a web-compatible database search or multiple alignment viewer. Bioinformatics 14:380–381. doi:10.1093/bioinformatics/14.4.380. 9632837

