# Supplementary figures and images for: Interferon-Responsive Genes Are Targeted during the Establishment of Human Cytomegalovirus Latency
Source: mBio. 2019 Dec 3;10(6):e02574-19. doi: 10.1128/mBio.02574-19 (PMC6890990; doi:10.1128/mBio.02574-19)

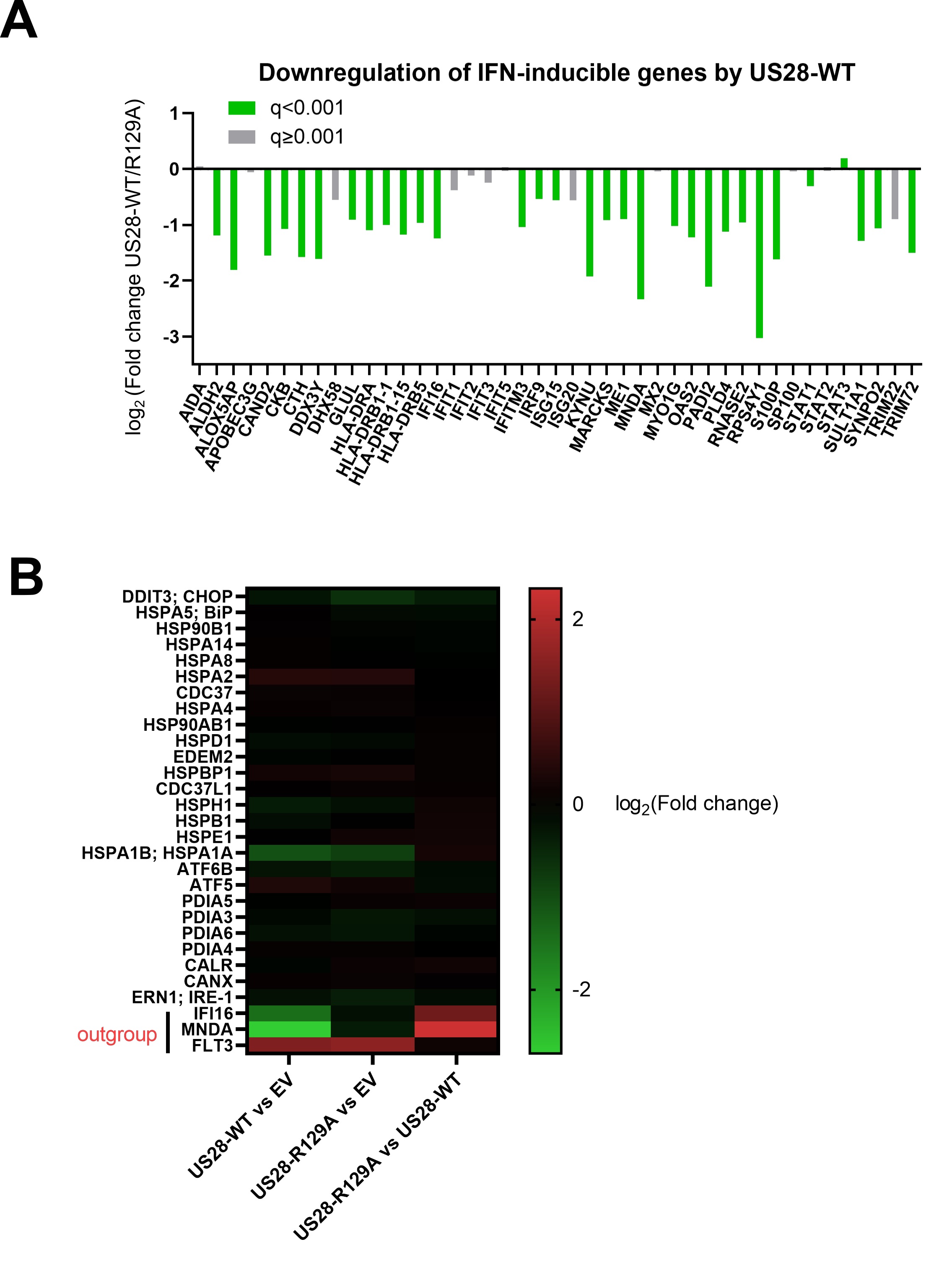

Supplement: FIG S1 [file mBio.02574-19-sf001.jpg]

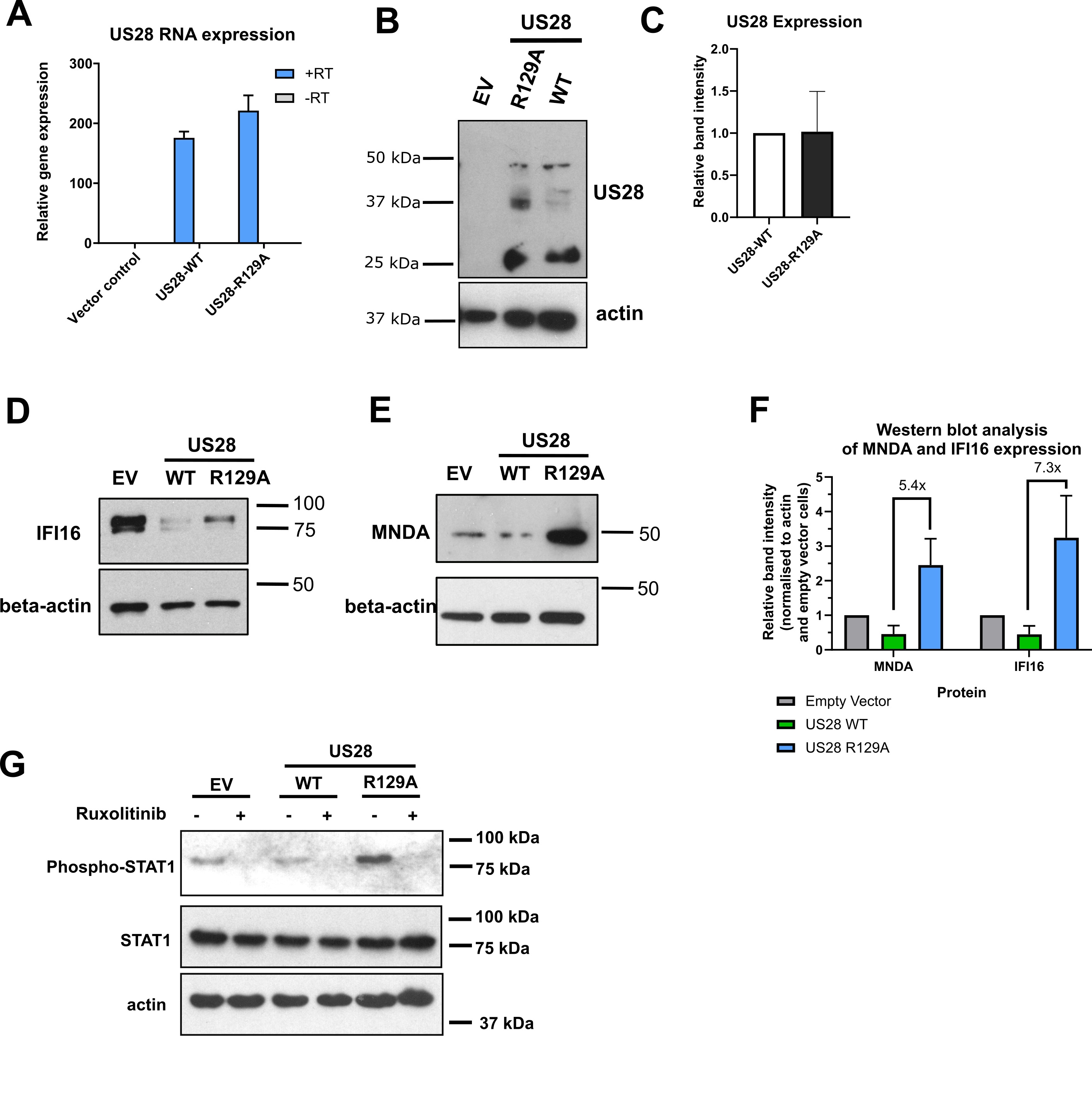

Supplement: FIG S2 [file mBio.02574-19-sf002.jpg]

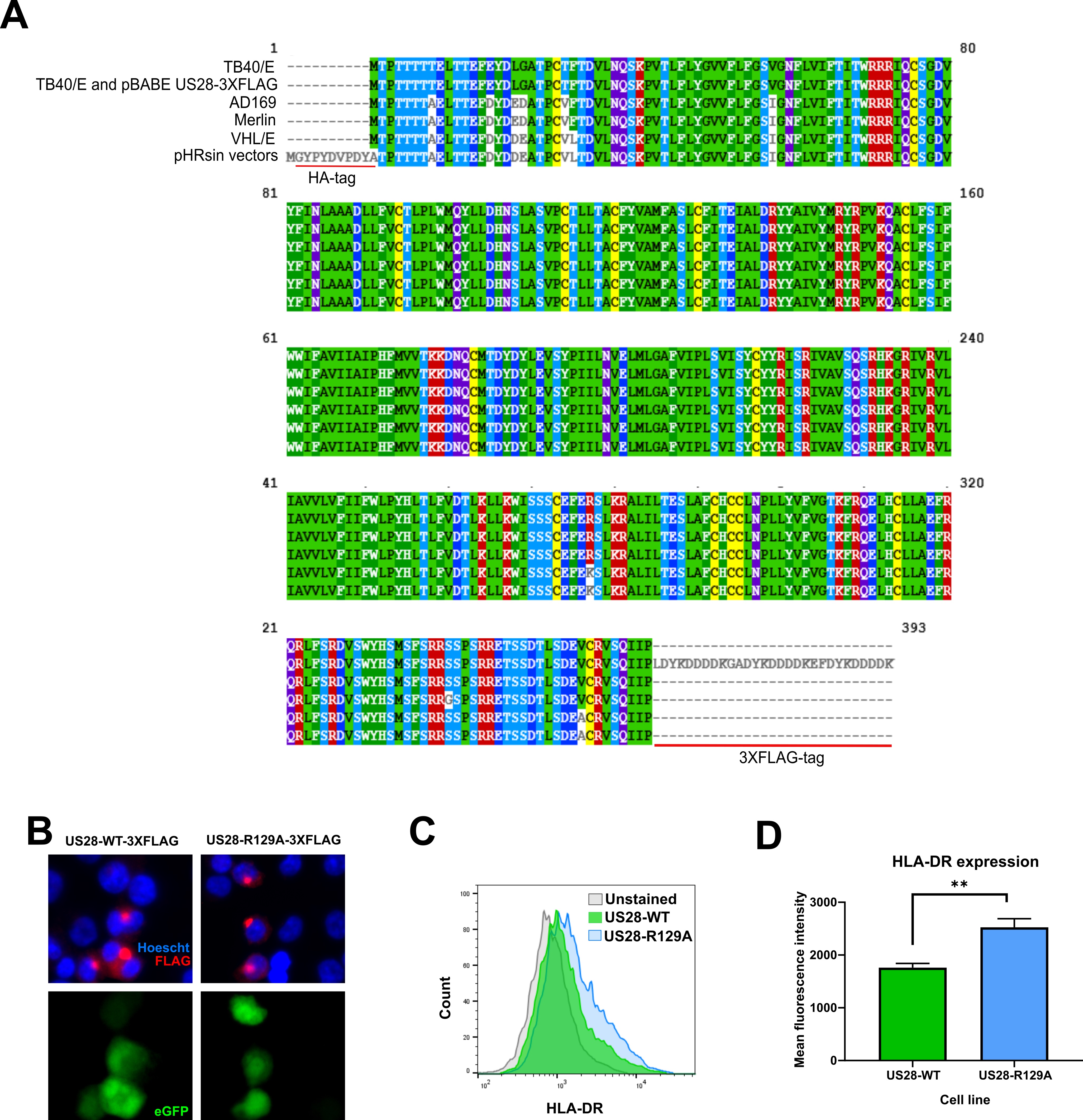

Supplement: FIG S3 [file mBio.02574-19-sf003.jpg]

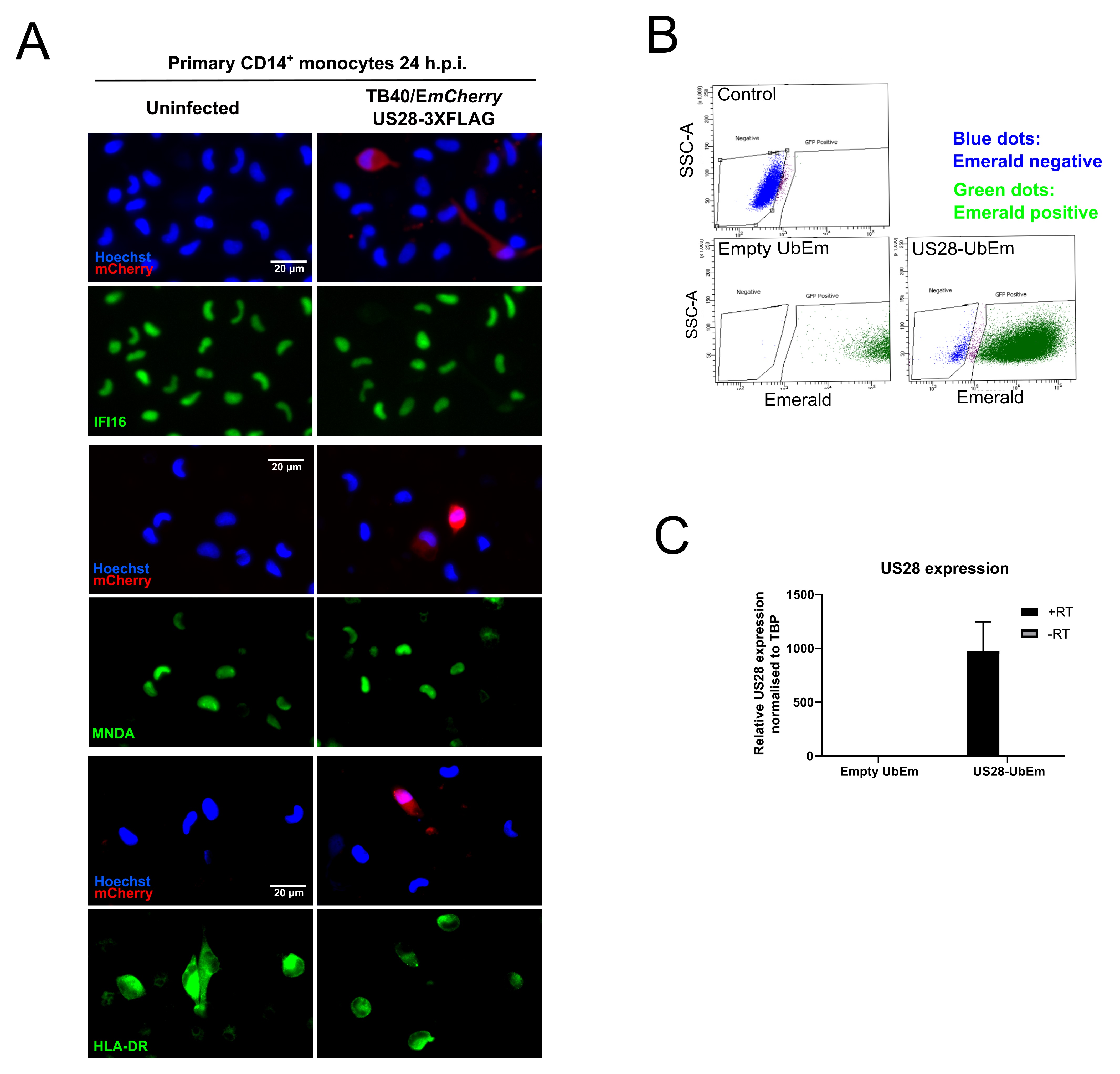

Supplement: FIG S4 [file mBio.02574-19-sf004.jpg]
