# Supplementary material for: Interferon-Responsive Genes Are Targeted during the Establishment of Human Cytomegalovirus Latency
Source: mBio. 2019 Dec 3;10(6):e02574-19. doi: 10.1128/mBio.02574-19 (PMC6890990; doi:10.1128/mBio.02574-19)
Supplement: TABLE S1 [file mBio.02574-19-st001.pdf]

MS settings for IP samples

Method Summary

Method Settings

Method Duration (min): **97**

Global Parameters

Ion Source

Ion Source Type: **NSI**
Spray Voltage: **Static**
Positive Ion (V): **2100.00**
Negative Ion (V): **600.00**

Positive Ion

Positive Ion	
Time (min)	Voltage (V)

Negative Ion

Negative Ion	
Time (min)	Voltage (V)

Sweep Gas (Arb): **0**
Ion Transfer Tube Temp (°C): **275**
Use Ion Source Settings from Tune: **False**

Experiment 1

Start Time (min): **0**
End Time (min): **97**
Cycle Time (sec): **3**

Master Scan:

MS OT

Detector Type: **Orbitrap**
Orbitrap Resolution: **120000**
Mass Range: **Normal**
Use Quadrupole Isolation: **False**
Scan Range (m/z): **375-1500**
RF Lens (%): **60**
AGC Target: **4.0e5**
Maximum Injection Time (ms): **50**
Microscans: **1**
Data Type: **Profile**
Polarity: **Positive**
Source Fragmentation: **Disabled**
Use EASY-IC™: **False**

Scan Description:

Filters:

MIPS

Monoisotopic Peak Determination: **Peptide**

Charge State

Include charge state(s): **2-5**
Include undetermined charge states: **False**
Include charge states 25 and higher: **False**

Dynamic Exclusion

Exclude after n times: **1**
Exclusion duration (s): **60**
Mass Tolerance: **ppm**
Low: **10.00**
High: **10.00**
Exclude Isotopes: **True**
Perform dependent scan on single charge state per precursor only: **True**

Intensity

Filter Type: **Intensity Threshold**
Intensity Threshold: **5.0e3**

Data Dependent

Data Dependent Mode: **Cycle Time**
Time between Master Scans (sec): **3**

Scan Event Type 1:

Scan:

ddMS^2 IT HCD

Isolation Mode: **Quadrupole**
Use Isolation m/z Offset: **False**
Activation Type: **HCD**
HCD Collision Energy (%): **35**
Stepped Collision Energy: **False**
Detector Type: **Ion Trap**
Scan Range Mode: **Auto: m/z Normal**
Ion Trap Scan Rate: **Rapid**
First Mass (m/z): **110**
AGC Target: **5.0e3**
Inject Ions for All Available Parallelizable Time: **True**
Maximum Injection Time (ms): **300**
Microscans: **1**
Data Type: **Centroid**

Scan Description:

MS settings for TMT labelled samples

Method Summary

Method Settings

Method Duration (min): **190**

Global Parameters

Ion Source

Ion Source Type: **NSI**
Spray Voltage: **Static**
Positive Ion (V): **2100.00**
Negative Ion (V): **600.00**

Positive Ion

Positive Ion	
Time (min)	Voltage (V)

Negative Ion

Negative Ion	
Time (min)	Voltage (V)

Sweep Gas (Arb): **0**
Ion Transfer Tube Temp (°C): **275**
Use Ion Source Settings from Tune: **False**

Experiment 1

Start Time (min): **0**
End Time (min): **190**
Cycle Time (sec): **3**

Master Scan:

MS OT

Detector Type: **Orbitrap**
Orbitrap Resolution: **120000**
Mass Range: **Normal**
Use Quadrupole Isolation: **True**
Scan Range (m/z): **400-1400**
RF Lens (%): **60**
AGC Target: **2.0e5**
Maximum Injection Time (ms): **50**
Microscans: **1**
Data Type: **Profile**
Polarity: **Positive**
Source Fragmentation: **Disabled**
Use EASY-IC™: **False**

Scan Description:

Filters:

MIPS

Monoisotopic Peak Determination: **Peptide**

Charge State

Include charge state(s): **2-7**
Include undetermined charge states: **False**
Include charge states 25 and higher: **False**

Dynamic Exclusion

Exclude after n times: **1**
Exclusion duration (s): **90**
Mass Tolerance: **ppm**
Low: **10.00**
High: **10.00**
Exclude Isotopes: **True**
Perform dependent scan on single charge state per precursor only: **True**

Data Dependent

Data Dependent Mode: **Cycle Time**
Time between Master Scans (sec): **3**

Scan Event Type 1:

Scan:

ddMS^2 IT CID

Isolation Mode: **Quadrupole**
Use Isolation m/z Offset: **False**
Activation Type: **CID**
CID Collision Energy (%): **35**
Activation Q: **0.25**
Multistage Activation: **False**
Detector Type: **Ion Trap**
Scan Range Mode: **Auto: m/z Normal**
Ion Trap Scan Rate: **Rapid**
AGC Target: **5.0e3**
Inject Ions for All Available Parallelizable Time: **True**
Maximum Injection Time (ms): **70**
Microscans: **1**
Data Type: **Centroid**

Scan Description:

Filters:

Isobaric Tag Loss Exclusion

Reagent: **TMT**

Precursor Selection Range

Mass Tolerance: **Mass Range**
Mass Range (m/z): **400-2000**

Precursor Ion Exclusion

Exclusion mass width: **m/z**
Low:: **30.00**
High:: **5.00**

Data Dependent

Number of SPS Precursors: **10**

Scan Event Type 1:

Scan:

ddMS^3 OT HCD

MS^n Level: **3**
Synchronous Precursor Selection: **True**
Number of SPS Precursors: **10**
Use Isolation m/z Offset: **False**
Activation Type: **HCD**
HCD Collision Energy (%): **65**
Detector Type: **Orbitrap**
Scan Range Mode: **Define m/z range**
Orbitrap Resolution: **50000**
Scan Range (m/z): **100-1000**
AGC Target: **5.0e4**
Inject Ions for All Available Parallelizable Time: **False**
Maximum Injection Time (ms): **120**
Microscans: **1**
Data Type: **Profile**
Use EASY-IC™: **False**

Scan Description:
